# Double zero-tillage and foliar-P nutrition coupled with bio-inoculants enhance physiological photosynthetic characteristics and resilience to nutritional and environmental stresses in maize–wheat rotation

**DOI:** 10.3389/fpls.2022.959541

**Published:** 2022-09-15

**Authors:** M. N. Harish, Anil K. Choudhary, Ingudam Bhupenchandra, Anchal Dass, G. A. Rajanna, Vinod K. Singh, R. S. Bana, T. Varatharajan, Parkash Verma, Saju George, G. T. Kashinath, M. Bhavya, S. K. Chongtham, E. Lamalakshmi Devi, Sushil Kumar, Soibam Helena Devi, Tshering Lhamu Bhutia

**Affiliations:** ^1^Division of Agronomy, ICAR–Indian Agricultural Research Institute, New Delhi, India; ^2^ICAR–Indian Institute of Horticultural Research, Farm Science Centre, Gonikoppal, India; ^3^Division of Crop Production, ICAR–Central Potato Research Institute, Shimla, India; ^4^ICAR–KVK, Tamenglong, ICAR Research Complex for NEH Region, Manipur Centre, Manipur, India; ^5^ICAR–Directorate of Groundnut Research, Regional Station, Anantapur, India; ^6^ICAR–Central Research Institute for Dryland Agriculture, Hyderabad, India; ^7^Agronomy Section, ICAR–National Dairy Research Institute, Karnal, India; ^8^Department of Agronomy, Mahatma Phule Krishi Vidyapeeth, Rahuri, India; ^9^Department of Agronomy, KSN University of Agricultural and Horticultural Sciences, Shivamogga, India; ^10^Multi Technology Testing Centre and Vocational Training Centre, CAEPHT, CAU, Ranipool, India; ^11^ICAR–Research Complex for North Eastern Region, Sikkim Centre, Tadong, India; ^12^Department of Crop Physiology, Assam Agricultural University, Jorhat, India

**Keywords:** conservation agriculture, P-fertilization, photosynthetic rate, phosphorus saving, radiation-use efficiency, relative water content

## Abstract

Conventionally tilled maize–wheat cropping system (MWCS) is an emerging cereal production system in semi-arid region of south-Asia. This system involves excessive tillage operations that result in numerous resource- and production-vulnerabilities besides impeding environmental-stresses. Likewise, phosphorus is a vital nutrient that limits crop growth and development. It’s a matter of great concern when ∼80% of Indian soils are low to medium in available-P due to its sparing solubility, resulting in crop stress and low yields. Hence, crop productivity, photosynthetic parameters and resilience to nutritional and environmental stresses were assessed in a MWCS using four crop-establishment and tillage management (CETM) practices [FBCT-FBCT (Flat bed-conventional tillage both in maize and wheat); RBCT-RBZT (Raised bed-CT in maize and raised bed-zero tillage in wheat); FBZT-FBZT (FBZT both in maize and wheat); PRBZT-PRBZT (Permanent raised bed-ZT both in maize and wheat)], and five P-fertilization practices [P_100_ (100% soil applied-P); P_50_+2FSP (50% soil applied-P + 2 foliar-sprays of P through 2% DAP both in maize and wheat); P_50_+PSB+AM-fungi; P_50_+PSB+AMF+2FSP; and P_0_ (100% NK with no-P)] in split-plot design replicated-thrice. The results indicated that double zero-tilled PRBZT–PRBZT system significantly enhanced the grain yield (6.1; 5.4 t ha^–1^), net photosynthetic rate (Pn) (41.68; 23.33 μ mol CO_2_ m^–2^ s^–1^), stomatal conductance (SC) (0.44; 0.26 mol H_2_O m^–2^ s^–1^), relative water content (RWC) (83.3; 77.8%), and radiation-use efficiency (RUE) (2.9; 2.36 g MJ^–1^) by 12.8–15.8 and 8.5–44.4% in maize and wheat crops, respectively over conventional tilled FBCT–FBCT. P_50_+PSB+AMF+2FSP conjugating soil applied-P, microbial-inoculants and foliar-P, had significantly higher Pn, SC, RUE and RWC over P_100_ besides saving ∼34.7% fertilizer-P under MWCS. P_50_+PSB+AMF+2FSP practice also had higher NDVI, PAR, transpiration efficiency and PHI over P_100_. Whereas lower stomatal limitation index (Ls) was observed under PRBZT–PRBZT system as compared to the conventional FBCT–FBCT system indicating that P is the limiting factor but not stomata. Hence, optimum P supply through foliar P-fertilization along with other sources resulted in higher grain yield by 21.4% over control. Overall, double zero-tilled PRBZT–PRBZT with crop residue retention at 6 t/ha per year, as well as P_50_+PSB+AMF+2FSP in MWCS, may prove beneficial in enhancing the crop productivity and, thereby, bolstering food security in semi-arid south-Asia region.

## Introduction

Achieving food security globally without damaging the environment and meeting the day-to-day human needs is one of the vital objectives of sustainable agriculture under the aegis of UN’s Sustainable Development Goals (Harish et al., 2022). In post-green revolution era, there have been enormous gains in crop yield that have brought food security besides boosting global economic growth, especially in India (Singh et al., 2011; Choudhary et al., 2018; Daisy and Giridhara, 2021). However, extensive tillage combined with conventional farming practices has resulted in increased soil erosion and gradual degradation of the resource base in current agriculture systems (Sharma and Singh, 2014). It is essential to replace conventional farming methods with location-specific climate-resilient agricultural methods to maintain farm production and profitability, to enhance farm livelihoods, to protect natural resources, and to reduce farm and environmental threats (Paul et al., 2014; Pooniya et al., 2015; Varatharajan et al., 2019a,b). Conservation agriculture (CA) appears to be a promising climate-resilient and resource-saving agricultural production strategy that aims for long-term agricultural production while also safeguarding the natural resources and the environment (Suraj and Behera, 2014; Faiz et al., 2022). CA aims to maximize agricultural yields and farm incomes while minimizing the negative environmental effects of traditional agriculture (Parihar et al., 2016). Zero-tillage is essential nowadays due to reduction in the cost of cultivation, greater retention of soil moisture, decreased energy consumption, increased farm revenues, improved soil biophysical and chemical characteristics, carbon sequestration, fewer greenhouse gas emissions, and comprehensive natural resource conservation (Paul et al., 2014; Jakhar et al., 2018; Varatharajan et al., 2019b; Pooniya et al., 2022). MWCS is India’s 3rd largest cropping system, covering ∼1.8 million ha and accounting for 3% of the country’s total food production (Pooniya et al., 2015). Cereal crops such as maize and wheat deplete the system’s nutritional supply. As a result, each of these crops requires effective fertilizer management strategies in order to reach their full yield potential. The MWCS is recently being followed in the Indo-Gangetic plains region (IGPR), which is already facing numerous challenges, such as natural resource degradation, declining agricultural productivity, and environmental concerns (Paul et al., 2014; Pooniya et al., 2017, 2022).

Crop performance under drought stress conditions is an indicator of the ability of the plants to utilize the available moisture in an efficient manner. Thus, the response of the plants to drought can be precisely examined by finding the characteristics which are linked to drought tolerance and mitigation (Mwadzingeni et al., 2016). Relative water content (RWC) has also been advocated as a more important indicator of water status than other water potential indicators in drought situations (Ghahfarokhi et al., 2015). Lowering of RWC causes stomata to close, resulting in a reduction in photosynthetic activity (Maghsoudi et al., 2016). Drought resistance is thought to be mediated by high RWC, which is generated by better osmotic regulation or decreased tissue cell wall flexibility (Essahibi et al., 2018). RWC of the leaf is also a better indicator of transpiration rate (Maghsoudi et al., 2016). This depicts how well a plant can recover from stress, which in turn affects yield and yield stability (Stomph et al., 2020). By utilizing the sun’s energy in the carbon fixation process, the photosynthesis transforms the biochemical processes on the Earth. The Calvin cycle is optimized in the C_4_ pathway by a higher concentration of CO_2_ reacting to RuBisCO, resulting in higher biomass productivity in C_4_ plants compared to C_3_ plants (Chandrasekaran et al., 2019). This reduces photorespiration and improves the plant’s water and P usage (Reyna-Llorens and Hibberd, 2017). Because of their higher biomass output and more efficient water use, C_4_ plants are thought to be more tolerant to abiotic stress and better adapted to semi-arid environments like IGPR. They can, however, achieve the same photosynthetic rate as C_3_ plants while having a significantly smaller stomatal aperture and resulting in less water loss (Way et al., 2014).

In CA-based systems, plant nutrition is a critical aspect of crop management. After nitrogen, phosphorus is the 2nd most vital plant nutrient that is involved in energy storage, translocation and coupling (Kumar et al., 2014). Furthermore, the management of P is very critical in MWCS, as P controls the transfer of genes, photosynthesis, respiration, reproduction, root development, blooming, fruiting, seed production, and seed output in plants (Sharon and Siobhan, 2016). Hence, P bioavailability to plants in an appropriate amount and form is highly essential. Yet, in most arable soils, P supply to vegetation is a serious limitation, having an average efficiency of only 10–20% (Kumar et al., 2014). The bulk of added P in acid Alfisol may react with iron (Fe) and aluminum (Al) ions to produce inaccessible Fe and Al hydroxyl phosphates (Kumar et al., 2016; Patra et al., 2022). In alkaline soils, applied-P again reacts with Ca and Mg ions to form Ca and Mg phosphates (Fang et al., 2018). Furthermore, farmers with limited resources cannot employ the necessary P levels due to high cost of P-fertilizers (Singh et al., 2022). In this context, the mutualistic arbuscular mycorrhizal fungi (AMF) found on the root endosphere of numerous plants, may assist in lowering the water and nutrient stresses and promoting the sustained plant growth (Porcel et al., 2012; Chandrasekaran et al., 2019). Alongside, P-fertilization will have affirmative impact on root growth which may further help in efficient use of other essential nutrients, leading to better crop yields while ameliorating the environmental stresses. The foliar-P nutrition can be another noble intervention to supply the P nutrition at critical crop growth stages besides escaping the P-fixation in the alkaline and acid soils. Many researchers have also enumerated the benefits of the foliar nutrition in various crops (Ankit et al., 2022; Bana et al., 2022; Dass et al., 2022).

Hence, it was hypothesized that the conservational agriculture and foliar P-fertilization coupled with bio-inoculants may be helpful in enhancing the physiological photosynthetic characteristics of the crop plants besides enabling them to cope-up the nutrient and environmental stresses under harsh semi-arid climates. Thus, it was planned to devise efficient P management strategies like foliar P-fertilization with bio-inoculants in the CA based crop management under MWCS so as to study their influence on crop productivity, photosynthesis, radiation-use efficiency (RUE), instantaneous water-use efficiency and P-use efficiency for sustaining the food and nutritional security under semi-arid IGPR.

## Materials and methods

### Experimental site and climate

**FIGURE 1:**
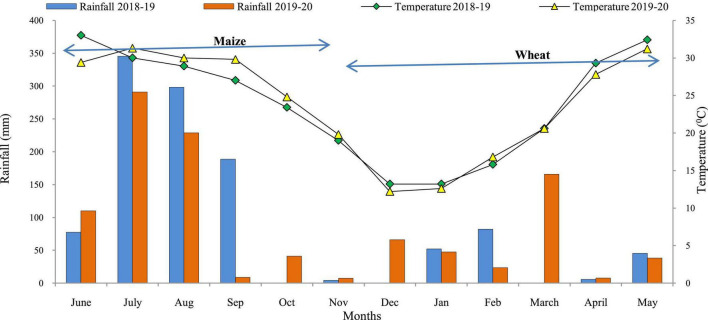
Monthly rainfall and mean temperature during crop growing seasons (2018–2020). Source: Agro-meteorological observatory, Division of Agricultural Physics, ICAR-IARI, New Delhi.

A field experiment was conducted during *Kharif* and *Rabi* seasons of 2018–19 and 2019–20 at Experimental Farm of ICAR-Indian Agricultural Research Institute, New Delhi, India [Latitude 28° 63’ N; Longitude 77° 15’ E; Altitude 228.6 m] under MWCS. The experimental site is located in a semi-arid region with Typic Ustochrepts sandy-loam alluvial soil. The experimental region has a subtropical climate with hot, dry summers and chilly winters, and it is located in the agro-climatic zone ‘Trans-Gangetic plains.’ The amount of precipitation received, mean monthly maximum and minimum temperature during the study period are depicted in Figure 1. The average annual precipitation was ∼650 mm, with 80 percent coming from the ‘South-West Monsoon’ and the rest coming from ‘Western Disturbances’ between December and February. A total of 976.1 and 884.2 mm of rainfall was received during first and second year of cropping cycle, respectively. The hottest months are May and June, with average monthly maximum temperatures ranging from 40 to 46°C, while the coldest months are December and January, with average minimum temperatures ranging from 2 to 6.2°C. The average annual evaporation was 850 mm. The soil in the experimental field has pH 8.0, SOC 0.421%, available–N 137.9 kg ha^–1^, available–P 12.9 kg ha^–1^, and available–K 302.8 kg ha^–1^ (Table 1).

**TABLE 1:** Physico-chemical and biological properties of the soil at the start of experiment.

	Particulars	Values	Methods followed
**I**	**Mechanical analysis**		
	1. Sand (%)	64.90	Hydrometer method (Bouyoucos, 1962)
	2. Silt (%)	21.03	
	3. Clay (%)	14.07	
	Textural class	Sandy- loam	
**II**	**Physical Properties**		
	1. Bulk density (Mg m^–3^)	1.57	Veihmeyer and Hendrickson (1948)
**III**	**Chemical properties**		
	Soil organic carbon (%)	0.42	Walkley and Black method (Jackson, 1973)
	Available N (kg ha^–1^)	137.9	Alkaline permanganate method (Subbiah and Asija, 1956)
	Available P (kg ha^–1^)	12.9	Olsen’s method (Olsen et al., 1954)
	Available K (kg ha^–1^)	302.8	Flame photometer method (Jackson, 1973)
	pH (1/2.5 soil: water ratio)	8.0	Beckman’s pH meter (Piper, 1965)
	EC (dSm^–1^) (1/2 soil: water ratio)	0.46	Richards (1954)
**IV**	**Biological properties**		
	Soil microbial biomass carbon (μg SMBC g soil^–1^)	183.36	Nunan et al. (1998)
	Dehydrogenase activity (μg TPF g soil^–1^ day^–1^)	28.33	Casida et al. (1964)
	Alkaline phosphatase activity (μg PNP g soil^–1^ hr^–1^)	185.35	Tabatabai and Bremner (1969)
	Acid phosphatase activity (μg PNP g soil^–1^ hr^–1^)	29.21	Tabatabai and Bremner (1970)
	Phosphorus solubilizing bacteria (cfu 10^4^/g of dry soil)	12.92	Dilution plate technique (Pikovskaya, 1948)

### Experimental details

The experiment comprised a total of 20-treatment combinations which included 4 main-plot treatments, i.e., CETM practices [M1: FBCT–FBCT (Flat bed–conventional tillage both in maize and wheat); M2: RBCT–RBZT (Raised bed–conventional tillage in maize and raised bed–zero tillage in wheat); M3: FBZT–FBZT (Flat bed–zero tillage both in maize and wheat); M4: PRBZT–PRBZT (Permanent raised bed–zero tillage both in maize and wheat)], and 5 P–fertilization practices in sub-plots [S1: P100 (100% P as basal); S2: P50+2FSP {50% P as basal (P50)+2 foliar sprays of phosphorus (2FSP) as DAP (2%) at knee-high stage (KHS) and pre-tasseling stage (PTS) in maize and tillering stage (TS) and pre-flowering stage (PFS) in wheat}; S3: P50+PSB+AMF {P50+PSB+AM-fungi(AMF)}; S4: P50+PSB+AMF+2FSP (P50+PSB+AMF+2FSP at KHS and PTS in maize, and at TS and PFS in wheat); S5: P0 {100% N and K with no-P (P0) as control}] which was laid-out in split-plot design replicated thrice (Table 2). In current investigation, the plot size was set to 5.0 m × 4.2 m, i.e., 21 m^2^ for both the maize and wheat. After sowing the subsequent crops of maize and wheat, crop residues from the previous season’s wheat and maize harvests were applied at the rate of 3 t ha^–1^, respectively to all the ZT-plots with the exception of CT-plots by uniformly spreading on the field to conserve the soil moisture and to curb the weed growth.

**TABLE 2:** Description of crop-establishment and tillage management (CETM) practices and P-fertilization practices in maize–wheat cropping system (MWCS).

Treatments	Crop establishment methods		Tillage management practices	
	Maize	Wheat	Maize	Wheat
**Main-plot treatments: CETM practices**					

**M_1_**	Flat-bed	Flat-bed	Conventional tillage	Conventional tillage
**M_2_**	Raised-bed	Raised-bed	Conventional tillage	Zero-tillage
**M_3_**	Flat-bed	Flat-bed	Zero-tillage	Zero-tillage
**M_4_**	Permanent raised-bed	Permanent raised-bed	Zero-tillage	Zero-tillage

**Sub-plot treatments: P-fertilization practices**					

**Treatments**	**Soil applied-P**	**Foliar-P**	**Microbial inoculants**	

**S_1_**	P_100_	**−**	**−**	
**S_2_**	P_50_	2 FSP	**−**	
**S_3_**	P_50_	**−**	PSB+AMF	
**S_4_**	P_50_	2 FSP	PSB+AMF	
**S_5_**	P_0_	**−**	**−**	

M_1_, flat bed–conventional tillage (FBCT) both in maize and wheat; M_2_, raised bed–CT (RBCT) in maize and RB–zero tillage (RBZT) in wheat; M_3_, FBZT both in maize and wheat; M_4_, permanent raised bed–ZT (PRBZT) both in maize and wheat. S_1_-P_100_, 100% P as basal; S_2_-P_50_ + 2FSP, 50% P as basal (P_50_) + 2 foliar sprays of phosphorus (2FSP) as DAP (2%) at knee-high stage (KHS) and pre-tasseling stage (PTS) in maize and at tillering stage (TS) and pre-flowering stage (PFS) in wheat; S_3_-P_50_ + PSB + AMF, P_50_ + PSB + AM-fungi (AMF); S_4_-P_50_ + PSB + AMF + 2 FSP, P_50_ + PSB + AMF + 2FSP at KHS and PTS in maize and at TS and PFS in wheat; S_5_-P_0_, 100% N and K with no P (P_0_) as control.

### Crop management

Maize (hybrid PMH-1) was seeded on 12th and 9th July at a spacing of 60 cm × 25 cm by seed-drill at the rate of 20 kg seeds ha^–1^ with fertilizer N: P_2_O_5_: K_2_O @ 150: 60: 40 kg ha^–1^ and harvested on 29th and 24th October, respectively during *Kharif* 2018 and 2019. Wheat [Variety HD-2967] was seeded on 13th and 10th November with a spacing of 22.5 cm (inter-row) using seed-drill @ 100 kg ha^–1^ with fertilizer N: P_2_O_5_: K_2_O (120: 60: 40 kg ha^–1^) and harvested on 15th and 17th April, respectively during Rabi 2018–19 and 2019–20. As a basal dose, whole-K and treatment-specific fertilizer-P were administered. N supplied in 3-equal splits for maize (basal, top-dressed at KHS and PTS) and for wheat (½ basal, ¼ CRI and ¼ PFS). Foliar P-fertilization was done at KHS and PTS in maize and at TS and PFS in wheat using 2% DAP (18% N and 46% P_2_O_5_) in 750 L water ha^–1^. In case of ZT plots, Glyphosate (1.0 kg a.i.ha^–1^) was applied a week before planting, following the harvest of the previous crop, to provide an early weed-free situation for crop emergence. Except for treatments, both the crops were grown using standard crop management practices (Rana et al., 2014).

### Phenological stages

At each growth stage, the number of days required (after sowing) for the occurrence of a particular growth stage in maize and wheat was recorded. Days taken to 50% tasseling and silking were recorded for maize, while days taken to 50% blooming were recorded for wheat.

### Normalized difference vegetation index and soil and plant analysis development values

The digital NDVI values were measured for each treatment in maize and wheat crops using a hand-held crop sensor, the Green Seeker (TMX-2050 handheld GreenSeeker™, United States), by holding the instrument 45 cm above the fully opened leaf of the crop canopy of the respective crops and walking along the crop rows. The SPAD (soil and plant analysis development) meter was used to determine the chlorophyll index. At the blooming stage, the 10 tagged plants used for plant height observations in maize and wheat were employed to take SPAD readings. Three different SPAD readings were taken from each plant at the flowering stage from the uppermost fully opened healthy leaves by holding the leaf in between the sensor of SPAD meter (Minolta SPAD-502 Plus chlorophyll meter; United States). After that, the readings were averaged to generate a single SPAD value from each plot.

### Photosynthetically active radiation interception

The PAR interception of a given treatment was calculated using a crop analyzer (LP-80 AccuPAR device). By subtracting the value of penetrated PAR at the bottom of the crop canopy from incident PAR at the top, the net intercepted PAR was calculated. This device measures both incident and transmitted radiation. From this intercepted solar radiation, PAR was calculated using the below mentioned formula:


(1) $$\begin{array}{c} \mathrm{PAR}\,\mathrm{interception}=\\ \mathrm{Incident}\,\mathrm{solar}\,\mathrm{radiation}-\mathrm{transmitted}\,\mathrm{solar}\,\mathrm{radiation} \end{array}$$

### Photosynthetic characteristics

The photosynthetic parameters were measured using Infrared Gas Analyzer (IRGA) LI-6400XT Portable Photosynthetic System (LI-COR, Lincoln, NE, United States) during the flowering stage of maize and wheat during both the years of experimentation, averaged later. The measured photosynthetic data included the net photosynthetic rate (Pn, μ mol CO_2_ m^–2^ s^–1^), stomatal conductance (Sc, mol H_2_O m^–2^ s^–1^), intercellular CO_2_ concentration (Ci, μ mol CO_2_ mol^–1^) and leaf transpiration rate (Tr, m mol H_2_O m^–2^ s^–1^). Each measurement was taken five times on the youngest fully developed leaf of each tagged plant during the hours of 9:00–11:00 a.m. on a windless and sunny day. In the leaf chamber, the PAR, CO_2_ concentration, flow rate, and temperature were set at 1700 mol (photon) m^2^ s^–1^, 380 mol CO_2_ mol^–1^, 500 mol s^–1^, and 30°C (Maize) and 25°C (Wheat), respectively.

The transpiration efficiency was measured as:


(2) $$\begin{array}{c} \mathrm{Transpiration}\,\mathrm{efficiency}\,\left(\mu \,\text{m}\,\text{o}\,\text{l}\,\text{C}\,{\text{O}}_{2}\,{\left(m\,\mathrm{mol}\,H_{2}\,\text{O}\right)}^{-1}\right)=\\ \frac{\mathrm{Photosynthetic}\,\mathrm{rate}}{\mathrm{Transpiration}\,\mathrm{rate}} \end{array}$$

### Grain and biomass yield

Both maize and wheat crop were harvested manually from net-plot area 1260 m^2^ (21 m^2^ × 60 plots) of the experimental field, to determine the yield. The harvested maize cobs were shelled plot by plot, the grains were cleaned, and the seeds were sun-dried until the seed moisture reached 14%. After harvesting, the wheat crop was sun-dried till 12% seed moisture; threshed plot-wise using Pullman thresher and grains cleaned were obtained. Grain and straw yield were determined using standard procedures as suggested by Rana et al. (2014).

### Stomatal limitation index

The contribution of the stomatal aperture to leaf photosynthesis is measured by the stomatal limitation index (Ls). The higher the Ls value, the stronger the stomatal aperture restricting plant photosynthesis. The Ls were determined as follows (Ma et al., 2021):


(3) $$L\,s=\left(1-\frac{C\,i}{C\,a}\right)\times 100\%$$

Where, Ci is the intercellular CO_2_ concentration, μmol CO_2_/mol; Ca is the external CO_2_ concentration, which in this study was set to 380 μmol CO_2_/mol in this study.

The partial closure of leaf stomata and a decrease in photosynthetic activity of mesophyll cells were two factors that contributed to the decrease in Pn value. The former was referred to as the stomatal factor, while the latter was referred to as the non-stomatal factor. The change in direction of Ci and Ls, according to Xu (1997)’s assessment technique, was a solid indicator of the decline of Pn value. The stomatal factor was the main cause of the decreasing Ci and growing Ls values, whereas the non-stomatal component was the main source of the increasing Ci and decreasing Ls values.

### Radiation-use efficiency

Global solar radiation (MJ m^–2^) was calculated using the Angstrom formula (coefficients *a* = 0.32, *b* = 0.46) from daily strong sunshine hours measured at the IARI Agro-meteorological observatory (Samanta et al., 2019). Daily incident photosynthetically active radiation (PAR) was calculated by multiplying global radiation by a factor of 0.48. Daily incoming PAR data were multiplied by matching daily fraction IPAR (fIPAR) values to calculate daily Intercepted-PAR (IPAR). During the crop growth season, IPAR was accumulated on a daily basis. In maize and wheat, the RUE (g MJ^–1^) was predicted to be under:


(4) $$\text{RUE}=\frac{\mathrm{Total}\,\mathrm{biomass}\,\left(\text{g}\right)}{\text{IPAR}\,\left(\text{MJ}\right)}$$

### Relative water content

The RWC in the leaves is calculated as suggested by Barr and Weatherley (1962) and expressed in %:


(5) $$\text{RWC}\left(\%\right)=\frac{\left(\text{FW}-\text{DW}\right)}{\left(\text{TW}-\text{DW}\right)}\times 100$$

Where, RWC = relative water content in the leaves (%), FW = wet weight (g), DW = dry weight (g) and TW = full weight (g).

### Phosphorus indices

The P concentration in grains and straw was evaluated using the vanado-molybdo-phosphoric acid yellow color technique on a UV-VIS spectrophotometer at 420 nm wavelength (Rana et al., 2014). Plant P content was measured in percent age, and P uptake (kg/ha) was calculated using the formula below.


(6) $$\begin{array}{c} P\,\mathrm{uptake}\,\left(\mathrm{kg}\,{\mathrm{ha}}^{-1}\right)\,\mathrm{in}\,\mathrm{grain}/\text{straw}=\\ \frac{\%P\mathrm{in}\mathrm{grain}/\text{straw}\times \mathrm{grain}/\mathrm{straw}\mathrm{yield}\left(\mathrm{kg}{\mathrm{ha}}^{-1}\right)}{100}\, \end{array}$$

Total uptake of P (kgha^–1^) P uptake in grain + P uptake in straw

The following formula was used to determine phosphorus harvest index (PHI) for plant (Kumar et al., 2017) and expressed as %:


(7) $$\text{P}-\text{HI}=\frac{{\text{P}}_{\text{g}}}{{\text{P}}_{\text{t}}}$$

Where,

P_*g*_ = Total P uptake by grain; Pt = Total P uptake (grain + straw).

The P content per unit leaf area (P_*area*_) was calculated using the following formula and expressed as mg cm^–2^:


(8) $${\text{P}}_{\text{area}}=\frac{\text{LTPA}}{\text{LA}}$$

Where, P_*area*_ denotes P content per unit leaf area,

LTPA = amount of total P leaf accumulation (mg plant^–1^),

LA = total leaf area (cm^2^ plant^–1^).

### Root dry weight

At the flowering stage of maize and wheat, root samples were gathered to estimate root dry weight. The roots were gathered using a conventional procedure after a 30 cm^3^ volume of soil was dug open from the base of the plant stem as the center (Rana et al., 2014). The roots were then rinsed in standing water using sodium hexameta phosphate to remove any soil that had stuck to them (Agarwal and Sharma, 2002). Before obtaining readings, the roots were removed from the stems and air-dried. After that, the roots were sun-dried, then oven-dried for 48 h at 60–65*^o^*C, and the dry weights of the roots were recorded in a digital electronic scale.

### Statistical analysis

SPSS statistical software was used to conduct all statistical analyses (version 22.0; United States). To establish the statistical significance of each parameter, the data were subjected to analysis of variance. To analyze the significant differences between the CETM and P-fertilization techniques, a simple *t*-test was calculated (5 percent level of significance). To compare the means of variables between the CETM and P-fertilized plots, Duncan’s multiple range test (DMRT) was performed. The Pearson’s correlation matrix was used to determine the correlations (*p* < 0.05 and *p* < 0.01) between several photosynthetic indices, RUE, RWC, root dry weight, grain yield, P content, and its uptake. The heatmap was created using the Ward-algorithmic cluster analysis (CA) approach, with the squared Euclidean distance utilized to separate various groupings, clustering the identical CETM and P-fertilized plots, photosynthetic indices, and P-nutrient content and absorption. A heatmap is a two-dimensional representation of a data matrix, with discrete cells represented as colored frames proportional to their locus and a color grade. A cubicle’s color is proportional to its location on a color scale. The organization of the rows and columns is examined using hierarchical cluster analysis of both rows and columns. Ward’s hierarchical clustering results are represented by a dendrogram, which is a heat map graph (Strauss and Maltitz, 2017). Cluster analysis can also be used to group together disparate photosynthetic metrics.

The PCA was used to determine the connection between the various factors, as well as the association between selected photosynthetic parameters and grain yield. PCA is a statistical approach for converting a set of potentially linked data into principal components, which are uncorrelated variables (PC). It helps to improve the interpretation of data results and significantly reduces the procedure (Esdras et al., 2017). The PCs of a dataset are defined as linear combinations of variables that account for the most variance in the collection. Varimax rotation (Gotelli and Ellison, 2004) was employed to maximize the sum of the variance of the factor coefficients. The fundamental goal of PCA is to reduce the dimensionality of a dataset and limit information loss. PCs with high eigen values best describe system variance. As a result, only PCs with eigen values of 1 (Kaiser, 1960) were kept. Component scores, also known as factor scores and loadings, are used to represent the retrieved results of a PCA (Wold et al., 1987).

Biplot is a visual classification tool for multivariate datasets that allows you to find correlations between the variance-covariance structure of the variables, the values of observations on variables, and the Euclidean distances between observations in multi-dimensional space (Alkan et al., 2015). The associations between the response variables (photosynthetic indices) and crop yield were also evaluated using multiple linear multiple regression (SLMR) approaches. It’s a statistical model that explains how independent and dependent variables interact. It is an iterative strategy for selecting predictor variables for inclusion in a regression model (Campbell, 2001). Depending on the approach, the ideal combination of independent variables that best fits the dependent variable is identified by gradually adding or eliminating the one variable that has the greatest impact on the residual sum of squares (Rawlings, 1998). The forwarding selection and backward elimination methods are combined in the stepwise regression method.

Multiple linear regression was assessed using the following formula:

Y=iβ+0βx1+i⁢1βx2+i⁢2βpx+i⁢pε

Where, I = n observations,

Y_*i*_ = predicted variable,

x_*i*_ = explanatory variables,

β_0_ = y intercept (constant term), i.e., the value of y when both x_*i*_ and x_2_ are 0.

β_1_ and β_2_ = the regression coefficients that describe the change in y as a function of a one-unit change in x_*i1*_ and x_*i2*_, respectively.

βp = each explanatory variable’s slope coefficients.

ε = the model’s error term (also known as the residuals).

## Results

### Crop phenological stages

The effect of CETM and P–fertilization practices on the number of days taken to reach blooming stage in maize and wheat under the MWCS is given in Supplementary Table 1. In maize, phenological stages such as days taken to 50% tasseling and 50% silking were significantly not affected by the CETM practices. FBZT–FBZT and PRBZT–PRTZT took less number of days 52.4 and 51.2 to reach 50% tasseling in 2018 and 2019, respectively. PRBZT–PRBZT plots took fewer days to attain 50% silking (58.7 and 56.4 days) and 50% flowering (99.8 and 99.9 days) for maize and wheat during 2018–19 and 2019–20, respectively. However FBCT–FBCT practice required a maximum number of days to attain 50% tasseling, silking and flowering during both the years. Under P–fertilization practices, the respective minimum days taken to 50% tasseling (50.5; 49.7 days), 50% silking (56.5; 55.8 days), and 50% flowering (99.8; 99.9 days) were observed under P_50_+PSB+AMF+2FSP practice which was followed by P_50_+2FSP, P_100_, P_50_+PSB+AMF. No P fertilization (P_0_) took the maximum number of days for 50% tasseling, 50% silking, and 50% flowering in the current study. Between years no such variation was observed for the above parameters in the present study.

### Normalized difference vegetation index, soil and plant analysis development values and photosynthetically active radiation interception

**FIGURE 2:**
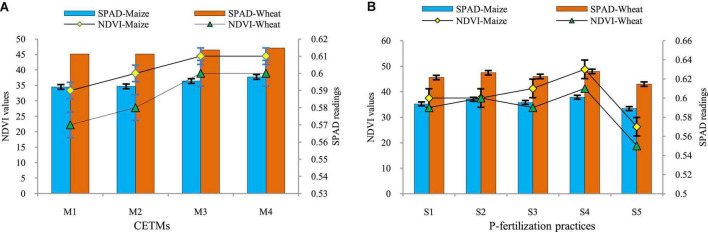
Effect of crop establishment and tillage management (CETM) **(A)** and P-fertilization practices **(B)** on NDVI, SPAD at flowering stage of maize and wheat under MWCS. *The vertical bars represent LSD_0.05_ values*. M_1_, flat bed–conventional tillage (FBCT) both in maize and wheat; M_2_, raised bed–CT (RBCT) in maize and RB–zero tillage (RBZT) in wheat; M_3_, FBZT both in maize and wheat; M_4_, permanent raised bed–ZT (PRBZT) both in maize and wheat. S_1_-P_100_, 100% P as basal; S_2_-P_50_ + 2FSP, 50% P as basal (P_50_) + 2 foliar sprays of phosphorus (2FSP) as DAP (2%) at knee-high stage (KHS) and pre-tasseling stage (PTS) in maize and at tillering stage (TS) and pre-flowering stage (PFS) in wheat; S_3_-P_50_ + PSB + AM-fungi (AMF); S_4_-P_50_ + PSB + AMF + 2 FSP at KHS and PTS in maize and at TS and PFS in wheat; S_5_-P_0_, 100% N and K with no-P (P_0_) as control.

The influence of both CETM and P–fertilization practices on NDVI, chlorophyll content as well as PAR interception at the flowering stage of both maize and wheat crops under MWCS is given in Table 3 and Figure 2. All the above parameters were significantly influenced under CETM and P–fertilization practices during both the years. Among CETM practices, the highest NDVI value of 0.61 and 0.60; SPAD value of 37.72 and 47.04 and PAR of 1341.5 and 1522.6 μ mol (photon) m^2^ s^–1^ was exhibited with PRBZT–PRBZT followed by FBZT–FBZT, RBCT–RBZT and least was observed with FBCT–FBCT [1170.6 and 1221.5 μ mol (photon) m^2^ s^–1^], respectively for maize and wheat in the current study. Overall, PRBZT–PRBZT practice resulted in higher PAR by ∼12.2 and 10.2%, respectively for maize and wheat over FBCT–FBCT practice, respectively.

**TABLE 3:** Influence of crop-establishment and tillage management (CETM) and P-fertilization practices on internal CO_2_ concentration, stomatal limitation index, PAR interception and relative water content at flowering stage as well as radiation-use efficiency of maize and wheat under MWCS.

Treatments	Intercellular CO_2_ conc. (μ mol CO_2_ mol^–1^)		Stomatal limitation index (%)		PAR Interception [μ mol (photon) m^2^s^–1^]		Radiation-use efficiency (g MJ^–1^)		Relative water content (%)	
					
	Maize	Wheat	Maize	Wheat	Maize	Wheat	Maize	Wheat	Maize	Wheat
**Crop-establishment and tillage management (CETM)**										
FBCT–FBCT	203.4c	236.4c	46.9a	38.4a	1196.0c	1382.0b	2.57b	1.92c	72.0b	71.7c
RBCT–RBZT	229.5*b*	275.5*b*	39.0ab	27.6bc	1256.0bc	1444.1ab	2.78ab	1.93bc	79.9a	74.1b
FBZT–FBZT	254.1ab	308.1a	32.0b	19.8cd	1308.1ab	1486.8a	2.83a	2.22a	82.6a	76.4ab
PRBZT–PRBZT	276.9a	330.9a	24.3c	13.8d	1341.5a	1522.6a	2.90a	2.36a	83.3a	77.8a
**P-fertilization practices**										
P_100_	231.9	278.7	38.8a	27.4	1217.4d	1446.6*a*b	2.65*b*c	2.08bc	76.5ab	73.7bc
P_50_+2FSP	245.7	292.5	34.1ab	23.9	1302.2bcd	1501.0a	2.78ab	2.11abc	79.7ab	74.8abc
P_50_+PSB+AMF	234.2	280.9	37.4ab	26.7	1277.7*cd*	1459.3a	2.91a	2.19ab	79.9ab	76.5ab
P_50_+PSB+AMF+2FSP	244.3	291.1	34.6ab	23.5	1461.2a	1569.3a	2.93a	2.24a	83.1a	78.8a
P_0_	248.8	295.6	33.0b	23.0	1118.5*e*	1318.3b	2.59c	1.98c	72.9b	71.1c

M_1_, flat bed–conventional tillage (FBCT) both in maize and wheat; M_2_, raised bed–CT (RBCT) in maize and RB–zero tillage (RBZT) in wheat; M_3_, FBZT both in maize and wheat; M_4_, permanent raised bed–ZT (PRBZT) both in maize and wheat. S_1_-P_100_, 100% P as basal; S_2_-P_50_ + 2FSP, 50% P as basal (P_50_) + 2 foliar sprays of phosphorus (2FSP) as DAP (2%) at knee-high stage (KHS) and pre-tasseling stage (PTS) in maize and at tillering stage (TS) and pre-flowering stage (PFS) in wheat; S_3_-P_50_ + PSB + AMF, P_50_ + PSB + AM-fungi (AMF); S_4_-P_50_ + PSB + AMF + 2 FSP, P_50_ + PSB + AMF + 2FSP at KHS and PTS in maize and at TS and PFS in wheat; S_5_-P_0_, 100% N and K with no P (P_0_) as control.

Application of P_50_+PSB+AMF+2FSP showed the highest NDVI value (0.63 and 0.64); SPAD value (37.72 and 47.04); and PAR [1341.5 and 1522.6 μ mol (photon) m^2^ s^–1^] among P–fertilization practices, followed by P_50_+2FSP, P_50_+PSB+AMF and P_100_ all of which were statistically significant over P_0_ treatment. Among P-fertilization practices, P_50_+PSB+AMF+2FSP treatment exhibited 30.6 and 19% higher PAR interception as compared to P_0_ treatment, respectively for maize and wheat. The interaction effect was observed to be non-significant for the above parameters in the present study.

### Net photosynthetic rate

**FIGURE 3:**
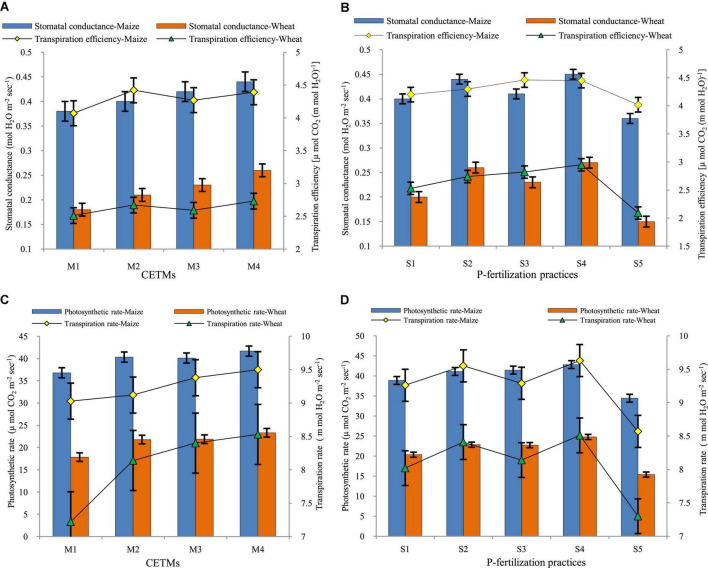
Effect of crop establishment and tillage management (CETM) and P-fertilization practices on **(A,B)** stomatal conductance and transpiration efficiency; **(C,D)** photosynthetic rate and transpiration rate at flowering stage of maize and wheat under MWCS. *The vertical bars represent LSD_0_._05_ values.* M_1_, flat bed–conventional tillage (FBCT) both in maize and wheat; M_2_, raised bed–CT (RBCT) in maize and RB–zero tillage (RBZT) in wheat; M_3_, FBZT both in maize and wheat; M_4_, permanent raised bed–ZT (PRBZT) both in maize and wheat. S_1_-P_100_, 100% P as basal; S_2_-P_50_ + 2FSP, 50% P as basal (P_50_) + 2 foliar sprays of phosphorus (2FSP) as DAP (2%) at knee-high stage (KHS) and pre-tasseling stage (PTS) in maize and at tillering stage (TS) and pre-flowering stage (PFS) in wheat; S_3_-P_50_ + PSB + AMF, P_50_ + PSB + AM-fungi (AMF); S_4_-P_50_ + PSB + AMF + 2 FSP, P_50_ + PSB + AMF + 2FSP at KHS and PTS in maize and at TS and PFS in wheat; S_5_-P_0_, 100% N and K with no P (P_0_) as control.

**FIGURE 4:**
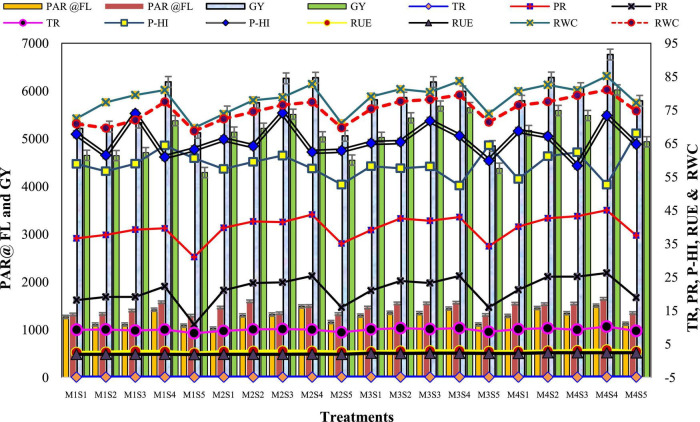
Effect of crop establishment and tillage management (CETM) and P-fertilization practices on photosynthetic indices of maize and wheat under MWCS (2-years’ av.). *The vertical bars represent the standard error of mean (SEM± at p ≤ 0.05)*. M_1_, flat bed–conventional tillage (FBCT) both in maize and wheat; M_2_, raised bed–CT (RBCT) in maize and RB–zero tillage (RBZT) in wheat; M_3_, FBZT both in maize and wheat; M_4_, permanent raised bed–ZT (PRBZT) both in maize and wheat. S_1_-P_100_, 100% P as basal; S_2_-P_50_ + 2FSP, 50% P as basal (P_50_) + 2 foliar sprays of phosphorus (2FSP) as DAP (2%) at knee-high stage (KHS) and pre-tasseling stage (PTS) in maize and at tillering stage (TS) and pre-flowering stage (PFS) in wheat; S_3_-P_50_ + PSB + AMF, P_50_ + PSB + AM-fungi (AMF); S_4_-P_50_ + PSB + AMF + 2 FSP, P_50_ + PSB + AMF + 2FSP at KHS and PTS in maize and at TS and PFS in wheat; S_5_-P_0_, 100% N and K with no P (P_0_) as control.

The CETM and P-fertilization practices under CA significantly influenced the Pn rate of both maize and wheat under MWCS (Figures 3, 4). The CA based treatment PRBZT–PRBZT resulted in significantly (*p* < 0.05) higher Pn of both maize (41.68 μ mol CO_2_ m^–2^ s^–1^) and wheat (23.33 μ mol CO_2_ m^–2^ s^–1^) as compared to other CETM practices during both years, respectively. Overall, PRBZT–PRBZT treatment showed improved Pn of both maize and wheat by ∼13.2 and 30.7%, over FBCT–FBCT treatment, respectively. Likewise, the influence of P–fertilization practices on Pn of maize and wheat differed significantly (*P* < 0.05) with the highest Pn of both maize (42.83 μ mol CO_2_ m^–2^ s^–1^) and wheat (24.77 μ mol CO_2_ m^–2^ s^–1^) over rest of the P-fertilization practices, respectively. P_50_+PSB+AMF+2FSP treatment exhibited ∼10.2 and 21.8% higher Pn as compared to P_100_ treatment, respectively in the current study.

### Transpiration rate

The amount of water transpired per unit area in a given period of time is referred to as the transpiration rate (TR). The TR of both maize and wheat crops is depicted pictorially in Figures 3, 4. Among CETM practices in MWCS, the highest TR was observed with PRBZT–PRBZT treatment (9.50; 8.53 m mol H_2_O m^–2^ s^–1^) where crop residue was retained @ 6 t ha^–1^ year^–1^ as compared to FBCT–FBCT practice which recorded the least TR (9.02; 7.22 m mol H_2_O m^–2^ s^–1^), respectively for maize and wheat in the current study. Overall, PRBZT–PRBZT treatment showed improved TR of both maize and wheat by ∼5.3 and 18.1%, over FBCT–FBCT treatment, respectively. The following trend of PRBZT–PRBZT > FBZT–FBZT > RBCT–RBZT > FBCT–FBCT was observed for TR in the present study. Among P-fertilization practices, the highest TR in maize (7.22 m mol H_2_O m^–2^ s^–1^) and wheat (7.22 m mol H_2_O m^–2^ s^–1^) was observed under P_50_+PSB+AMF+2FSP which was followed by P_50_+2FSP, P_50_+PSB+AMF, P_100_ and P_0_, respectively. P_50_+PSB+AMF+2FSP treatment exhibited ∼12.4 and 16.6% higher TR as compared to P_100_ treatment, respectively in the current study.

### Stomatal conductance and transpiration efficiency

Stomatal conductance (Gs) is a measure of stomatal opening and is proportional to photosynthesis and transpiration intensity. Gs was significantly influenced by both CETM and P-fertilization practices under CA based MWCS (Figure 3). The CA based treatment PRBZT–PRBZT resulted in significantly (*p* < 0.05) highest Gs (0.44; 0.26 mol H_2_O m^–2^ s^–1^), respectively in maize and wheat as compared to other CETM practices. Significantly higher transpiration efficiency (TE) was observed under PRBZT–PRBZT practice (4.39 μ mol CO_2_ (m mol H_2_O)^–1^) and (2.73 μ mol CO_2_ (m mol H_2_O)^–1^) over the rest of the P-fertilization practices, respectively for maize and wheat. The following trend of PRBZT–PRBZT > RBCT–RBZT > FBZT–FBZT > FBCT–FBCT was observed for transpiration efficiency (TE) in the present study. Overall, PRBZT–PRBZT treatment showed improved Gs of both maize and wheat by ∼15.7 and 44.4%, over FBCT–FBCT treatment, respectively. Among P–fertilization practices, Gs of maize and wheat differed significantly (*p* < 0.05) with the highest Gs (0.45; 0.27 mol H_2_O m^–2^ s^–1^) over the rest of the P-fertilization practices, respectively. The Gs in MWCS followed the trend of P_50_+PSB+AMF+2FSP > P_50_+2FSP > P_50_+PSB+AMF > P_100_ > P_0_ in current study. Likewise, the influence of P–fertilization practices on TE of maize and wheat differed significantly (*p* < 0.05) with the highest TE of both maize (4.45 μ mol CO_2_ (m mol H_2_O)^–1^) and wheat (2.95 μ mol CO_2_ (m mol H_2_O)^–1^) over rest of the P-fertilization practices, respectively.

### Grain and by-product yield

**FIGURE 5:**
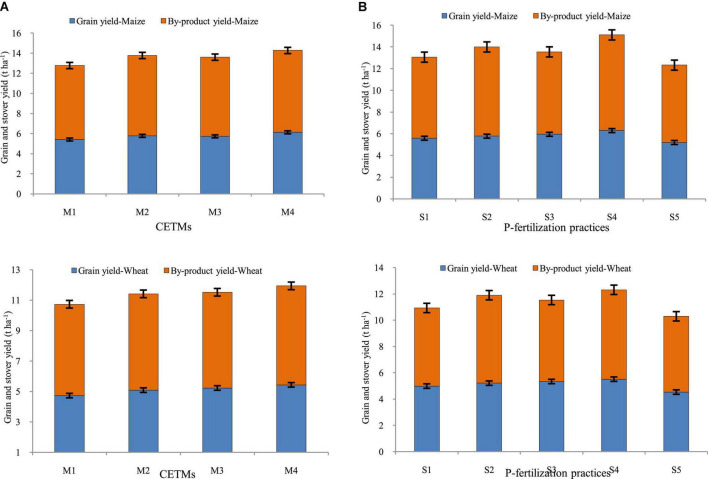
Effect of crop establishment and tillage management (CETM) and P-fertilization practices on productivity of maize **(A)** and wheat **(B)** crop under MWCS (2-years’ av.). *The vertical bars represent LSD_0.05_ values.* M_1_, flat bed–conventional tillage (FBCT) both in maize and wheat; M_2_, raised bed–CT (RBCT) in maize and RB–zero tillage (RBZT) in wheat; M_3_, FBZT both in maize and wheat; M_4_, permanent raised bed–ZT (PRBZT) both in maize and wheat. S_1_-P_100_, 100% P as basal; S_2_-P_50_ + 2FSP, 50% P as basal (P_50_) + 2 foliar sprays of phosphorus (2FSP) as DAP (2%) at knee-high stage (KHS) and pre-tasseling stage (PTS) in maize and at tillering stage (TS) and pre-flowering stage (PFS) in wheat; S_3_-P_50_ + PSB + AMF, P_50_ + PSB + AM-fungi (AMF); S_4_-P_50_ + PSB + AMF + 2 FSP, P_50_ + PSB + AMF + 2FSP at KHS and PTS in maize and at TS and PFS in wheat; S_5_-P_0_, 100% N and K with no P (P_0_) as control.

The crop productivity in terms of grain yield and stover/straw yield of both maize and wheat was influenced significantly by both CETM as well as P-fertilization practices under CA based MWCS (Figures 4, 5). The CA based treatment PRBZT–PRBZT with crop residue retention at 6 t/ha per year resulted in considerably (*p* < 0.05) higher grain yield of both maize (6.14 t ha^–1^) and wheat (5.44 t ha^–1^), stover yield of maize (8.15 t ha^–1^) and straw yield of wheat (6.50 t ha^–1^) in both the years, respectively. Overall, PRBZT–PRBZT treatment improved the maize and wheat productivity by ∼13.2 and 14.9% for grain and 10.7 and 8.3% for stover/straw yield over FBCT–FBCT treatment, respectively. Among P-fertilization practices, P_50_+PSB+AMF+2FSP treatment differed substantially (*p* < 0.05) from the rest of the P-fertilization practices, with greater grain yields of maize (6.30 t ha^–1^) and wheat (5.52 t ha^–1^), stover yield of maize (8.82 t ha^–1^) and straw yield of wheat (6.8 t ha^–1^) over rest of the P-fertilization practices, respectively (Figure 5). Likewise, the highest system productivity was recorded in P_50_+PSB+AMF+2FSP treatment (12.42 t ha^–1^) over rest of the treatments. Among P-fertilization practices, P_50_+PSB+AMF+2FSP treatment exhibited ∼4.5, 7.3, 11.6, and 21.4% higher system productivity compared to P_50_+PSB+AMF, P_50_+2FSP, P_100_, P_0_, respectively in current study. Hence, P_50_+PSB+AMF+2FSP conjugating soil applied-P, microbial-inoculants and foliar-P, had significantly higher crop yields over P_100_ besides saving ∼34.7% fertilizer-P under MWCS.

### Stomatal limitation index and intercellular CO_2_ concentration

The key indicators for evaluating the decline in Pn are the stomatal limitation index (Ls) and intercellular CO_2_ concentration (Ci). As a result of comparing Table 3, we hypothesized that Pn was significantly affected by Ci and Ls under CA based MWCS. Increasing Ls value and decreasing Ci value under CETM based FBCT–FBCT treatment explained that the stomatal factor was the chief cause for lower Pn among CETM practices. Among P-fertilization practices, P_0_ differed considerably (*p* < 0.05) with superior Ci values indicating that non-stomatal factor is the main cause of decline in Pn. The CETM and P-fertilization practices followed the trend of FBCT–FBCT > RBCT–RBZT > FBZT–FBZT > PRBZT–PRBZT and P_100_ > P_50_+PSB+AMF > P_50_+PSB+AMF+2FSP ≥ P_50_+2FSP > P_0_ for Ls, whereas Ci followed the trend of PRBZT–PRBZT > FBZT–FBZT > RBCT–RBZT > FBCT–FBCT and P_0_ > P_50_+2FSP > P_50_+PSB+AMF+2FSP > P_50_+PSB+AMF > P_100_ under CA based MWCS, respectively.

### Radiation-use efficiency and relative water content in leaves

Radiation-use efficiency was significantly higher in PRBZT–PRBZT practice (2.90; 2.36 g MJ^–1^) for maize and wheat, respectively as compared to other CETM practices under CA based MWCS (Figure 4). RUE was ∼12.8 and 22.9% higher under PRBZT–PRBZT treatment for maize and wheat over FBCT–FBCT practice, respectively. Among P-fertilization practices, P_50_+PSB+AMF+2FSP treatment differed considerably (*p* < 0.05) with superior RUE of maize (2.93 g MJ^–1^) and wheat (2.24 g MJ^–1^) over the rest of the P-fertilization practices, respectively (Table 3). The RUE followed the trend of P_50_+PSB+AMF+2FSP > P_50_+PSB+AMF > P_50_+2FSP > P_100_ > P_0_, respectively for P-fertilization treatments.

The results in Table 3 shows the effective impact of CETM practices and P-fertilization treatments on the leaf RWC both in maize and wheat plant. It is clear that the values of the RWC achieved the highest mean of 83.3 and 77.8% under CA based PRBZT–PRBZT practice as compared to the conventional tillage based FBCT–FBCT practice which reported a decrease in the water content of the leaves with a rate of 72.0 and 71.7% for maize as well as wheat, respectively. Among P-fertilization methods, P_50_+PSB+AMF+2FSP treatment differed appreciably (*p* < 0.05) with elevated RWC of maize (83.1%) and wheat (78.8%) over the rest of the P-fertilization practices, respectively (Figure 4). The RWC also followed the similar trend as that of RUE for P-fertilization practices with P_50_+PSB+AMF+2FSP treatment displayed ∼14 and 10.8% superior RWC as compared to P_0_ treatment, respectively for maize as well as wheat in the present research.

### Phosphorus concentration before and after foliar-P spray in maize and wheat plants

**FIGURE 6:**
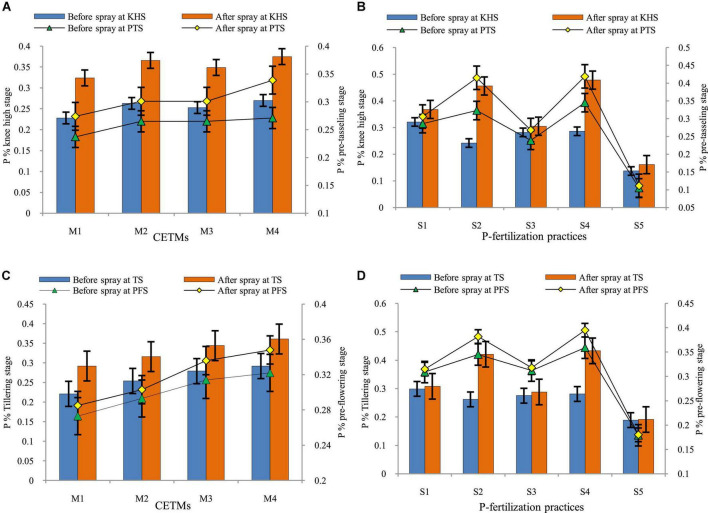
Effect of crop establishment and tillage management (CETM) and P-fertilization practices on phosphorus content before and after spray at knee-high (KHS) and pre-tasseling stage (PTS) of maize **(A,B)** and at tillering stage (TS) and pre-flowering stage (PFS) of wheat **(C,D)** under MWCS (2-years’ av.). *The vertical bars represent LSD_0.05_ values.* M_1_, flat bed–conventional tillage (FBCT) both in maize and wheat; M_2_, raised bed–CT (RBCT) in maize and RB–zero tillage (RBZT) in wheat; M_3_, FBZT both in maize and wheat; M_4_, permanent raised bed–ZT (PRBZT) both in maize and wheat. S_1_-P_100_, 100% P as basal; S_2_-P_50_ + 2FSP, 50% P as basal (P_50_) + 2 foliar sprays of phosphorus (2FSP) as DAP (2%) at knee-high stage (KHS) and pre-tasseling stage (PTS) in maize and at tillering stage (TS) and pre-flowering stage (PFS) in wheat; S_3_-P_50_ + PSB + AMF, P_50_ + PSB + AM-fungi (AMF); S_4_-P_50_ + PSB + AMF + 2 FSP, P_50_ + PSB + AMF + 2FSP at KHS and PTS in maize and at TS and PFS in wheat; S_5_-P_0_, 100% N and K with no P (P_0_) as control.

Crop-establishment and tillage management and P–fertilization methods influence the P concentration in maize and wheat, before and after the foliar–P spray (2% DAP) KHS and PTS of maize and TS and PFS of wheat under MWCS is represented graphically in Figure 6. The P content (%) before and after the foliar–P spray was significantly impacted by the CETM and P–fertilization practices; it was found to be higher in ZT based system (PRBZT–PRBZT, RBCT–RBZT) over respective CT based counterparts (FBZT–FBZT, FBCT–FBCT) for both before and after foliar–P spray at KHS and PTS with the highest magnitude under PRBZT–PRBZT. Among P–fertilization practices, significantly highest P content (0.321%) was obtained by applying the P_100_ at KHS before foliar–P spray, whereas application of P_50_+PSB+AMF+2FSP resulted in highest P content (0.478%) after foliar–P spray at KHS, and before and after foliar–P spray at PTS (0.345; 0.419%), respectively; followed by P_50_+2FSP, P_100_, P_50_+PSB+AMF and lowest under P_0_ during both years.

The critical perusal of data on P content (%) before and after foliar–P spray of DAP at TS and PFS of wheat was significantly impacted by the CETM and P–fertilization practices. It was found that P content was significantly higher under PRBZT–PRBZT followed by FBZT–FBZT and RBCT–RBZT which are statistically on par with each other while the lowest P content was found under FBCT–FBCT practice both before and after foliar–P spray at MTS and PFS in wheat under MWCS. Among P–fertilization practices, significantly highest P content (0.299%) was obtained under P_100_ at TS before foliar–P spray, whereas P_50_+PSB+AMF+2FSP resulted in the highest P content (0.433%) after foliar–P spray at TS (0.359; 0.395%) before and after foliar–P spray at PFS, respectively which was followed by P_50_+2FSP, P_100_, P_50_+PSB+AMF with lowest values under P_0_ during both the years of experimentation. Significant interaction effect was found for all crop stages except P% before foliar–P spray at TS wheat.

### Phosphorus content and uptake

**FIGURE 7:**
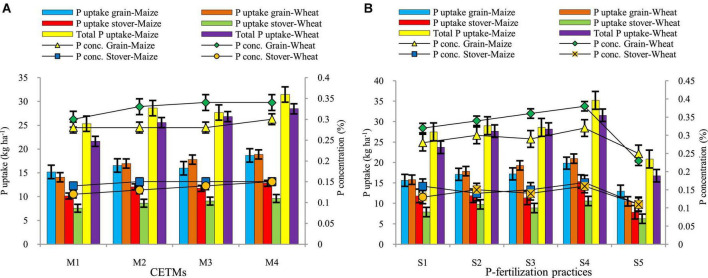
Effect of crop establishment and tillage management (CETM) **(A)** and P-fertilization practices **(B)** on P content and P uptake in grains and stover and total P uptake in maize and wheat under MWCS (2-years’ av.). *The vertical bars represent LSD_0.05_ values*. M_1_, flat bed–conventional tillage (FBCT) both in maize and wheat; M_2_, raised bed–CT (RBCT) in maize and RB–zero tillage (RBZT) in wheat; M_3_, FBZT both in maize and wheat; M_4_, permanent raised bed–ZT (PRBZT) both in maize and wheat. S_1_-P_100_, 100% P as basal; S_2_-P_50_ + 2FSP, 50% P as basal (P_50_) + 2 foliar sprays of phosphorus (2FSP) as DAP (2%) at knee-high stage (KHS) and pre-tasseling stage (PTS) in maize and at tillering stage (TS) and pre-flowering stage (PFS) in wheat; S_3_-P_50_ + PSB + AM-fungi (AMF); S_4_-P_50_ + PSB + AMF + 2FSP at KHS and PTS in maize and at TS and PFS in wheat; S_5_-P_0_, 100% N and K with no P (P_0_) as control.

Crop-establishment and tillage management and P–fertilization strategies have a considerable impact on P content in grain, straw, and its uptake in maize and wheat under MWCS are depicted graphically in Figure 7. Highest grain-P (0.30; 0.34%) and stover/straw-P (0.15; 0.15%) were observed under PRBZT–PRBZT treatment whereas lowest grain-P (0.28; 0.30%) and stover/straw-P (0.14; 0.12%) was observed in FBCT–FBCT in maize and wheat crop, respectively. The FBZT–FBZT and RBCT–RBZT displayed intermediate grain-P and straw-P content and were found to be on par with each other. In the current investigation, P content followed the PRBZT–PRBZT > FBZT–FBZT > RBCT–RBZT > FBCT–FBCT pattern. The highest uptake of P in grains (18.65; 18.85 kg ha^–1^), stover/straw-P uptake (12.78; 9.59 kg ha^–1^), and total P-uptake (31.44; 28.44 kg ha^–1^) were found under PRBZT–PRBZT treatment. Similarly, the P uptake followed the same trend as that of P content in grains and stover/straw as mentioned above.

Among P–fertilization practices, significantly highest grain-P (0.32; 0.38%) and stover/straw-P (0.17; 0.16%) were obtained by applying P_50_+PSB+AMF+2FSP with lowest grain-P (0.25; 0.23%) and straw-P (0.11; 0.11%) under P_0_ treatment, respectively for maize and wheat crop. The P uptake in grains (19.86; 20.97 kg ha^–1^), stover/straw (15.37; 10.61 kg ha^–1^) and total P uptake (35.22; 31.58 kg ha^–1^) were highest under P_50_+PSB+AMF+2FSP during both the years, respectively under maize and wheat crop with lowest values under P_0_ treatment in the current study. Maize and wheat crops under ZT based PRBZT–PRBZT and P_50_+PSB+AMF+2FSP exhibited ∼22.9; 34.1% and 26.9; 27.2% higher grain P-uptake, respectively over their respective counterpart treatments *viz*. FBCT–FBCT and P_100_. The interaction effect between CETM with P–fertilization practices for P-concentration as well as P-uptake both in grains and stover/straw was found significant during both the years of experimentation.

### Phosphorus harvest index and P content per unit leaf area

The effect of CETM practices and P–fertilization practices on P-harvest index (PHI) and P content per unit leaf area (PCPULA) is given in Table 4 and Figure 4. There was no significant effect of CETM practices on PHI, with the maximum PHI (59.89%) found under FBCT–FBCT for maize and the lowest PHI (66.45%) found under PRBZT–PRBZT for wheat, respectively. However, the PCPULA was significantly affected by CA based MWCS. Among CETM practices, the highest PCPULA was found with PRBZT–PRBZT (0.022 and 0.025 mg cm^–2^) followed by RBCT–RBZT and FBZT–FBZT while the lowest PCPULA was found under FBCT–FBCT (0.018 and 0.021 mg cm^–2^) practice, respectively in maize and wheat.

**TABLE 4:** Influence of crop-establishment and tillage management (CETM) and P-fertilization practices on P-harvest index (PHI), P content per unit leaf area (PCPULA), and root dry weight (RDW) of maize and wheat crops under MWCS (2-years’ av.).

Treatments	PHI (%)		PCPULA (mg cm^–2^)		RDW (g)	
			
	Maize	Wheat	Maize	Wheat	Maize	Wheat
**Crop-establishment and tillage management (CETM)**						
FBCT–FBCT	59.89a	65.45a	0.018*b*	0.021b	22.41d	4.42b
RBCT–RBZT	57.67a	65.96a	0.020ab	0.022ab	23.95bc	5.00a
FBZT–FBZT	58.15a	65.88a	0.018b	0.023ab	23.74c	5.03a
PRBZT–PRBZT	59.71a	66.45a	0.022a	0.025a	25.63a	5.08a
P**-fertilization practices**						
P_100_	57.19ab	66.94abc	0.019d	0.022b	24.68c	4.80bc
P_50_+2FSP	58.73*ab*	64.54cd	0.024b	0.026a	23.33d	4.73c
P_50_+PSB+AMF	60.18ab	69.54a	0.020cd	0.025ab	24.69bc	5.24ab
P_50_+PSB+AMF+2FSP	56.73*b*	66.00*bc*	0.029a	0.027a	26.26a	5.44a
P_0_	61.44a	62.66d	0.007e	0.013c	20.70e	4.21d

M_1_, flat bed–conventional tillage (FBCT) both in maize and wheat; M_2_, raised bed–CT (RBCT) in maize and RB–zero tillage (RBZT) in wheat; M_3_, FBZT both in maize and wheat; M_4_, permanent raised bed–ZT (PRBZT) both in maize and wheat. S_1_-P_100_, 100% P as basal; S_2_-P_50_ + 2FSP, 50% P as basal (P_50_) + 2 foliar sprays of phosphorus (2FSP) as DAP (2%) at knee-high stage (KHS) and pre-tasseling stage (PTS) in maize and at tillering stage (TS) and pre-flowering stage (PFS) in wheat; S_3_-P_50_ + PSB + AMF, P_50_ + PSB + AM-fungi (AMF); S_4_-P_50_ + PSB + AMF + 2 FSP, P_50_ + PSB + AMF + 2FSP at KHS and PTS in maize and at TS and PFS in wheat; S_5_-P_0_, 100% N and K with no P (P_0_) as control.

During both years of the trial, the P–fertilization techniques had a considerable impact on the PHI and PCPULA. In maize highest PHI (61.44%) was found under P_0_ treatment and in wheat highest PHI (66.94%) was recorded under P_100_ treatment, respectively. Significantly highest PCPULA (0.029 and 0.027 mg cm^–2^) were obtained by applying P_50_+PSB+AMF+2FSP treatment and the lowest PCPULA (0.007 and 0.013 mg cm^–2^) under P_0_ treatment, respectively in maize and wheat. There was a significant interaction effect between CETM and P–fertilization practices for PHI in current study.

### Root dry weight at flowering stage

The experimental results showed that the CETM and P–fertilization approaches had a substantial effect on root dry weight in maize and wheat as given in Table 4. Highest root dry weight (25.43; 25.83 g) was found under PRBZT–PRTZT plots with the lowest root dry weight (25.63; 5.08 g) being obtained under FBCT–FBCT plots for maize and wheat, respectively. During both years of the current study, the root dry weight under CETM procedures followed the pattern of PRBZT–PRBZT > RBCT–RBZT ≥ FBZT–FBZT > FBCT–FBCT. Among P–fertilization practices, P_50_+PSB+AMF+2FSP resulted in considerably increased root dry weight (26.26; 5.44 g) for maize and wheat, respectively. In general, the root dry weight under P–fertilization practices followed the trend of P_50_+PSB+AMF+2FSP > P_50_+PSB+AMF > P_100_ > P_50_+2FSP > P_0_ in the current study. Interaction effect between CETM and P–fertilization practices was found significant for root dry weight during the second year of experimentation only.

### Correlation, clustered heatmap, principal components analysis and stepwise linear multiple regression analysis

To determine the link between the variables like photosynthetic indices, RUE, RWC, root dry weight, grain-P uptake and grain yield under CETM and P-fertilization practices, Pearson’s correlation coefficient analysis was used (Table 5). The findings revealed a significantly significant positive correlation (*p* < 0.01) between grain yield with grain-P uptake (*r* = 0.856; 0.930), PAR interception (*r* = 0.826; 0.836), photosynthetic rate (*r* = 0.860; 0.898), transpiration rate (*r* = 0.782; 0.832), transpiration efficiency (*r* = 0.731; 0.765), stomatal conductance (*r* = 0.798; 0.868), root dry weight (*r* = 0.832; 0.774) and RWC (*r* = 0.842; 0.934) and they were significantly correlated to each other, respectively in maize and wheat. However, maize grain yield showed considerable positive association with RUE (*r* = 0.860; 0.898), while wheat grain yield showed with P content per unit leaf area (*r* = 0.753). Similarly, grain-P uptake had a highly considerable positive association (*p* < 0.01) with PAR interception (*r* = 0.708; 0.783), photosynthetic rate (*r* = 0.798; 0.919), transpiration rate (*r* = 0.700; 0.802), transpiration efficiency (*r* = 0.711; 0.816), stomatal conductance (*r* = 0.709; 0.901), and RWC (*r* = 0.799; 0.811) and they were significantly correlated to each other, respectively in maize and wheat. However grain-P uptake had a substantially significant positive association (*p* < 0.01) with root dry weight (*r* = 0.729) and P content per unit leaf area (*r* = 0.801). Photosynthetic rate too resulted in a substantially significant positive association (*p* < 0.01) with PAR interception (*r* = 0.796; 0.814), transpiration rate (*r* = 0.871; 0.862), transpiration efficiency (*r* = 0.897; 0.918), stomatal conductance (*r* = 0.870; 0.884), root dry weight (*r* = 0.833; 0.780), P content per unit leaf area (*r* = 0.800; 0.878), and RWC (*r* = 0.896; 0.893), respectively in maize and wheat. While photosynthetic rate of maize was found to correlate positively with RUE (*p* < 0.01 and *r* = 0.860). On the other hand, P-harvest index, intercellular CO_2_ concentration and stomatal limitation index showed no significant association with any of the parameters.

**TABLE 5:** Pearson’s correlation coefficients matrix (r) between photosynthetic indices, RUE, RWC, RDW, and P-uptake in grains (PUG) of maize and wheat.

	PUG	PAR@FL	PR	TR	TE	SC	RDW	PHI	ICO_2–_C	SLI	PCPULA	RUE	RWC	GY
**In maize**														
**PUG**	***1.000***	0.708	***0.798***	0.700	***0.711***	***0.709***	0.691	0.209	0.292	−0.318	0.704	0.680	***0.799***	***0.856***
**PAR@FL**	0.708	***1.000***	***0.769***	***0.788***	0.570	***0.782***	***0.712***	−0.224	0.375	−0.378	0.706	0.618	***0.802***	***0.826***
**PR**	***0.798***	***0.769***	***1.000***	***0.871***	***0.897***	***0.870***	***0.833***	−0.268	0.352	−0.361	***0.800***	***0.860***	***0.896***	***0.860***
**TR**	0.700	***0.788***	***0.871***	***1.000***	0.565	***0.913***	***0.807***	−0.339	0.303	−0.306	***0.740***	***0.722***	***0.871***	***0.782***
**TE**	***0.711***	0.570	***0.897***	0.565	***1.000***	0.632	0.675	−0.146	0.303	−0.316	0.676	***0.794***	***0.716***	***0.731***
**SC**	***0.709***	***0.782***	***0.870***	***0.913***	0.632	***1.000***	***0.778***	−0.310	0.483	−0.488	***0.755***	***0.761***	***0.863***	***0.798***
**RDW**	0.691	***0.712***	***0.833***	***0.807***	0.675	***0.778***	***1.000***	−0.357	0.272	−0.285	0.692	***0.728***	0.702	***0.832***
**PHI**	0.209	−0.224	−0.268	−0.339	−0.146	−0.310	−0.357	***1.000***	0.057	−0.086	−0.308	−0.170	−0.180	−0.085
**ICO2-C**	0.292	0.375	0.352	0.303	0.303	0.483	0.272	0.057	***1.000***	***−0.997***	0.119	0.455	0.438	0.439
**SLI**	−0.318	−0.378	−0.361	−0.306	−0.316	−0.488	−0.285	−0.086	***−0.997***	***1.000***	−0.114	−0.475	−0.441	−0.454
**PCPULA**	0.704	0.706	***0.800***	***0.740***	0.676	***0.755***	0.692	−0.308	0.119	−0.114	***1.000***	0.521	***0.716***	0.654
**RUE**	0.680	0.618	***0.860***	***0.722***	***0.794***	***0.761***	***0.728***	−0.170	0.455	−0.475	0.521	***1.000***	***0.803***	***0.751***
**RWC**	***0.799***	***0.802***	***0.896***	***0.871***	***0.716***	***0.863***	0.702	−0.180	0.438	−0.441	***0.716***	***0.803***	***1.000***	***0.842***
**GY**	***0.856***	***0.826***	***0.860***	***0.782***	***0.731***	***0.798***	***0.832***	−0.085	0.439	−0.454	0.654	***0.751***	***0.842***	***1.000***
**In wheat**														
**PUG**	***1.000***	***0.783***	***0.919***	***0.802***	***0.816***	***0.901***	***0.729***	0.406	0.343	−0.358	***0.801***	0.448	***0.881***	***0.930***
**PAR@FL**	***0.783***	***1.000***	***0.814***	***0.728***	***0.715***	***0.824***	***0.738***	0.153	0.469	−0.483	**0.685**	0.457	***0.837***	***0.836***
**PR**	***0.919***	***0.814***	***1.000***	***0.862***	***0.918***	***0.884***	***0.780***	0.257	0.416	−0.430	***0.878***	0.477	***0.893***	***0.898***
**TR**	***0.802***	***0.728***	***0.862***	***1.000***	**0.599**	***0.833***	**0.650**	0.318	**0.682**	**−0.689**	**0.633**	**0.647**	***0.823***	***0.832***
**TE**	***0.816***	***0.715***	***0.918***	**0.599**	***1.000***	***0.728***	***0.757***	0.163	0.118	−0.132	***0.894***	0.252	***0.762***	***0.765***
**SC**	***0.901***	***0.824***	***0.884***	***0.833***	***0.728***	***1.000***	**0.587**	0.348	0.482	−0.486	***0.801***	0.567	***0.830***	***0.868***
**RDW**	***0.729***	***0.738***	***0.780***	**0.650**	***0.757***	**0.587**	***1.000***	0.208	0.265	−0.283	**0.642**	0.330	***0.749***	***0.774***
**PHI**	0.406	0.153	0.257	0.318	0.163	0.348	0.208	***1.000***	−0.047	0.057	0.148	0.078	0.250	0.409
**ICO2-C**	0.343	0.469	0.416	**0.682**	0.118	0.482	0.265	−0.047	***1.000***	***−0.998***	0.193	***0.844***	**0.614**	0.507
**SLI**	−0.358	−0.483	−0.430	**−0.689**	−0.132	−0.486	−0.283	0.057	***−0.998***	***1.000***	−0.206	***−0.831***	**−0.629**	−0.519
**PCPULA**	***0.801***	**0.685**	***0.878***	**0.633**	***0.894***	***0.801***	**0.642**	0.148	0.193	−0.206	***1.000***	0.351	***0.718***	***0.753***
**RUE**	0.448	0.457	0.477	**0.647**	0.252	0.567	0.330	0.078	***0.844***	***−0.831***	0.351	***1.000***	**0.653**	0.555
**RWC**	***0.881***	***0.837***	***0.893***	***0.823***	***0.762***	***0.830***	***0.749***	0.250	**0.614**	**−0.629**	***0.718***	**0.653**	***1.000***	***0.925***
**GY**	***0.930***	***0.836***	***0.898***	***0.832***	***0.765***	***0.868***	***0.774***	0.409	0.507	−0.519	***0.753***	0.555	***0.925***	***1.000***

Boldfaced italics and boldfaced values indicate that correlation is positive and highly significant at p < 0.01% and p < 0.05% level of probability (2-tailed); the color assigned to a point in the heat map grid indicates the strength of a correlation between the soil parameters, and r-values correspond directly to the color codes ranging from green to yellow and red, respectively.

### Relationship between photosynthetic indices, radiation-use efficiency, relative water content with PUG in maize and wheat

**FIGURE 8:**
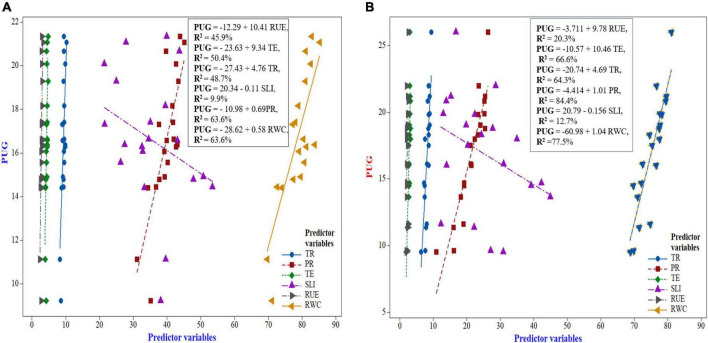
Regression response curve between photosynthetic indices and grain-P uptake of maize **(A)** and wheat **(B)**.

The regression response curve in indicating the contribution of diverse photosynthetic indices, RUE and RWC (predictor variables) to grain P uptake (response variable) of maize and wheat has been elucidated in Figure 8. The regression analysis between photosynthetic indices and grain P uptake of maize exhibited a linear and significant positive relationship. From the Figure 8, it can be deduced that among the photosynthetic indices, net photosynthetic rate and RWC had the highest coefficient of determination, *R*^2^ = 63.6%, indicating that net photosynthetic rate and RWC both bring about 63.6% of the variation of grain P uptake, and this rate is represented by the nearest dots to the linear line (Figure 8). Thus, a linear regression equation I was generated using the formula,

***y = a + bx***

where, **y** = response variable,

**a** = constant and **b** = regression slope and

**x** = independent predictor

**PUG** = −12.29 + 10.41RUE, ***R*^2^** = *45.9%* Equation I

This equation interpreted that with the increase in 1 unit (1 g MJ^–1^ of RUE), the PUG will increase at the rate of 10.41. Similarly, the following three equations were produced for the rest of yield attributes of FBPY as:

**PUG** = −23.63 + 9.34TE, ***R*^2^** = *50.4%* Equation II

**PUG** = −27.43 + 4.76TR, ***R*^2^** = *48.7%* Equation III

**PUG** = −20.34 − 0.11SLI, ***R*^2^** = *9.9%* Equation IV

**PUG** = −10.98 + 0.69PR, ***R*^2^** = *63.6%* Equation V

**PUG** = −28.62 + 0.58RWC, ***R*^2^** = *63.6%* Equation VI

So, amongst the generated equation, Equation V was the best fitting model and thus it can be inferred that net photosynthetic rate (PR) was the dominant photosynthetic indices attributed in improving the PUG followed by the sequence as RWC > RU > TE > TR > SLI.

Similarly, in respect of wheat crop, it can be inferred from Figure 8 that six fitting regression models were produced as follows:

**PUG** = −3.711 + 9.78RUE, ***R*^2^** = *20.3%* Equation VI

**PUG** = −10.57 + 10.46TE, ***R*^2^** = *66.6%* Equation II

**PUG** =−20.74 + 4.69TR, ***R*^2^** = *64.3%* Equation III

**PUG** = −4.414 + 1.01PR, ***R*^2^** = *84.4%* Equation IV

**PUG** = −20.79 – 0.156SLI, ***R*^2^** = *12.7%* Equation V

**PUG** = −60.98 + 1.04RWC, ***R*^2^** = *77.5%* Equation VI

Amongst the generated regression models, the equation IV was the best fitting model, wherein net photosynthetic rate (PR) was the dominant photosynthetic indices attributing to influencing the PUG followed by the sequence as RWC > TE > TR > RUE > SLI, respectively.

**FIGURE 9:**
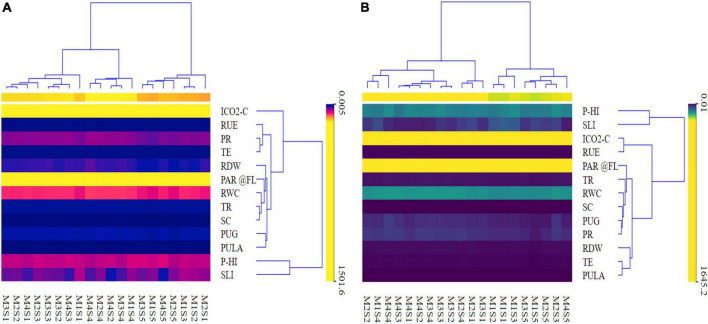
A clustering heat map depicting different photosynthetic indices, RUE, RWC, and grain-P uptake maize **(A)** and wheat **(B)**.

The result of heatmap clustering (Figure 9) is shown as the distance or similarity among the clustered rows or columns (variables) of the matrix, depending on the predetermined distance computed. The heatmap’s vertical row dendrogram (Figure 9) shows the similarity between rows of photosynthetic indices, RUE, RWC, root dry weight, and P indices, whereas the heatmap’s horizontal row dendrogram shows the CETM and P-fertilization plots. The heatmap (Table 3 and Figure 9) indicates that the hierarchical clustering approach retrieved four dominant vertical clusters of various plant indices, respectively for both maize and wheat and one outliner ICO_2_-C for maize. Correspondingly, four and five tangible clusters (Supplementary Table 2 and Figure 9) of CETM and P-fertilization practices were formed horizontally, respectively where M2S1 was the outlier for maize crop (Supplementary Table 2 and Figure 9). The RUE, PR, and TE; PHI and SLI comprised the first cluster in terms of photosynthetic and P indices and they were the least influencing variables impacting grain yield. Similarly, Cluster II included RDW, PAR@FL, RWC, TR, and SC; ICO_2_-C and RUE; while Cluster III had PUG and PCPULA, all of which had a minor impact on grain yield. Cluster IV is made up of PHI and SLI; RDW, TE, and PCPULA, which are the largest contributors to maize and wheat grain yields, respectively. The predictor variables involved in Cluster IV and the input order followed the trend of SLI > PHI in maize and RDW > PCPULA > TE in wheat, respectively (Supplementary Table 2 and Figure 9). Likewise, in the case of CETM and P-fertilization practices the Cluster I (MIS2, MIS3, M2S5, M4S5, M1S5, and M3S5; M4S5, M2S3, M2S5, M1S5, and M3S5), respectively in maize and wheat generally outperformed the other practices in respect of photosynthetic and P indices (Supplementary Table 2 and Figure 9).

**FIGURE 10:**
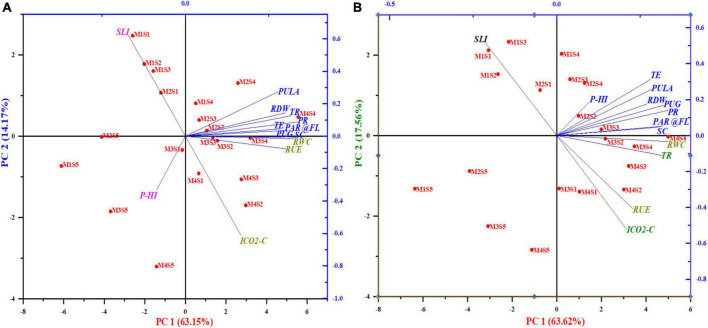
Two-dimensional graphical biplot depicting the loadings and score plot formed by principal components 1 and 2 of photosynthetic indices, RUE, RWC, RDW, and grain-P uptake of maize **(A)** and wheat **(B)**. Percentage values on PC1 and PC2 indicate the respective variance explained by the first two PCA axes.

Both the loading plots of photosynthetic features and the scores of the experimental locations were depicted in the principal component analysis (PCA) biplots (Figure 10 and Supplementary Table 3). The findings of PCA on maize and wheat plant features extracted three main components, each with eigenvalues >1 (Kaiser, 1960), which explained up to 86 and 88.89% of the total variability, respectively. The extracted three PCs in maize explained up to 63.13 percent of the overall variability of the data (PC1), 14.14% (PC2), and 8.73% (PC3). Similarly, the retrieved three PCs explained 63.62% (PC1), 17.50% (PC2), and 7.78% (PC3) of the overall variability of the data in wheat. In case of maize, the PCA revealed that PR, RWC and PUG were the most significant plant characters affecting the maize yield. Similarly, in respect of wheat, TE, PR and PCPULA are the vital plant parameters implicated in augmenting wheat yield. Biplot of PCA(maize); PC1 had substantial positive loadings on PR, followed by RWC and PUG, and they were strongly associated with each other, as the angle inside the variables of 0 or 1800 revealed a correlation of 1 or 1, respectively (Kohler and Luniak, 2005).

The score plot (Figure 10) in respect of wheat was performed to cluster the treatments based on similarities. The score plot (Figure 10) showing a plot of PC or partial least squares factors exhibited that the 1st quadrant consists of treatment M4S4, M2S4, MIS4, M2S3, M2S2 which were closely related to high loading variables and were closely associated with PC1. Similarly, the score plot showed that the 2nd and 3rd quadrant harboring plant characters that exhibited negative loadings and correlations with the rest of the quadrant exhibited the presence of quality parameters. While, the last quadrant had the PHI which did not impact the wheat grain yield.

Likewise, in respect of wheat (Figure 10), the score unveiled that the 1st quadrant embraced the vital treatments *viz.*, M3S3, M2S2, M2S4, M2S3, and M1S4 treatments which are related to positive high loadings implicated in leveraging wheat yield. The second quadrant accompanied the plant parameter SLI which showed negative correlation and loadings with the rest of the other parameters while the third quadrants did not harbor any particular treatment. The fourth quadrant was occupied by plant characters RUE, TR, RWC, and ICO_2_-C which had lower positive loadings as compared to 1st quadrant and they are much related to treatments *viz*., comparatively M3S2, M4S4, M3S4, M4S3, M4S2, M3S1, and M4S1, respectively.

The stepwise linear multiple regression (SLMR) analysis was used to remove redundant variables that were not linked to grain yield and to build interrelationships between the PAR interception, root dry weight, and P indices with maize and wheat yield. A best-fitting regression model was built using grain yield as a dependent attribute (response variable) and PAR interception, root dry weight, and P indices as independent attributes (predictor variables) (Eqs. 9, 10):


(9) $$\begin{array}{c} \mathrm{Maize}\,\mathrm{grain}\,\mathrm{yield}=1253+78.5\,\mathrm{PUG}+1.193\,\mathrm{PAR}\,@\,\mathrm{FL}\\ +80.9\,\mathrm{RDW}-12475\,PCPULA \end{array}$$

(10) $$\begin{array}{c} \mathrm{Wheatgrainyield}=2393+102.9\,\mathrm{PUG}+0.766\,\mathrm{PAR}\,@\,\mathrm{FL}\\ +42.26\,\mathrm{RDW}-18433\,PCPULA \end{array}$$

Where, PUG = grain P uptake, PAR@FL = Photosynthetically active radiation at flowering stage, RDW = root dry weight and PCPULA = P content per unit leaf area.

*Stepwise Selection of Terms: α to enter = 0.15, α to remove = 0.15.*

*R*^2^ = 89.48; 94.95%, respectively for maize and wheat.

Adjusted-*R*^2^ = 86.68; 93.61%, respectively for maize and wheat.

Predicted *R*^2^ = 82.07; 91.48%, respectively for maize and wheat.

Durbin-Watson Statistic = 1.63; 1.88, respectively for maize and wheat.

The results of SLMR analysis exhibited that independent attributes PUG, PAR@FL and RDW contributed an increment in the grain yield of maize and wheat to the tune of 73.26 and 56.55%; 9.73 and 10.83%; 4.80 and 25.03%, respectively; while PCPULA deterred the grain yield by 1.69 and 2.54% (Eqs. 9, 10 and Supplementary Table 4).

## Discussion

The number of days taken to 50% tasseling, 50% silking, and 50% flowering occurred early in ZT-based raised-bed (RB) or flat-bed (FB) plots owing to better plant establishment and vigorous growth due to temperature modulation, less water stagnation, more weed suppression and better nutrition under CA based CETMs over conventional tillage based CETMs (Varatharajan et al., 2019a,b; Faiz et al., 2022). Among P-fertilization practices, the combined action of PSB and AMF on P-solubilization as well as P-mobilization might have increased P-availability (Kumar et al., 2015; Harish et al., 2022). Furthermore, 2% DAP foliar application may have increased N and P uptake through maize leaves. As a result, in comparison to only usage of soil applied P_100_, the combined application of P_50_+PSB+AMF+2FSP displayed faster plant growth and development, and finally took lesser days for various phenological stages like 50% tasseling, 50% silking, and 50% flowering.

Photosynthetically active radiation interception in maize as well as wheat at flowering stage was similar and it was considerably higher in CA-based PRBZT–PRBZT compared to CT-based FBZT–FBZT (Table 3). This could be due to a greater leaf area index, which allows for better light absorption and photosynthesis. Improved planting geometry under raised-bed planting has harvested more solar energy due to good canopy development and crop growth (Varatharajan et al., 2019b). This resulted in increased plant photosynthesis and a competitive advantage over flat-bed sowing in terms of yield (Choudhary et al., 2018, 2020). The SPAD and NDVI values were also found to be considerably higher in CA based CETMs than CT based CETMs. SPAD and NDVI values were much higher in CA based CETMs due to an even crop canopy and strong leaf growth. However, crop growth, canopy cover, and leaf area were lower in CT plots, resulting in lower NDVI values in CT based CETMs than CA plots. Readings from the SPAD meter were found to be positively linked with leaf greenness, leaf NO_3_ status, photosynthesis and CO_2_ assimilation. The combined application of P_50_+PSB+AMF+2FSP resulted in higher PAR interception, SPAD, and NDVI values, which might be attributed to increased nutrient bio-availability (Kumar et al., 2015, 2016; Singh et al., 2022).

Among CETM practices, grain yield was higher where PRBZT both in maize and wheat crops as compared to the FBCT both in maize and wheat under MWCS during both years. Seed germination, early maize plant establishment, and growth were all improved in the PRBZT–PRTZT system due to crop residue retention (CRR), which prevented the formation of a hard crust on the soil surface (Choudhary et al., 2018, 2020), which is a common feature of IGPR alluvial soils (Paul et al., 2014; Choudhary et al., 2018). Similarly, ZT system might have reduced the soil and canopy temperature and evaporative losses in harsh summer season of semi-arid IGPR coinciding with an early vegetative phase of maize, resulting in improved growth and yield in maize (Varatharajan et al., 2019a,b). At the same time, improved crop residue decomposition might have improved SOC, SMBC, nutrient availability, soil moisture content, and biological activity, resulting in greater growth and productivity (Naresh et al., 2014; Choudhary et al., 2018, 2020; Bhupenchandra et al., 2022). Furthermore, in the ZT system, characteristics such as reduced resource competition, root aeration, and high fertilizer-use efficiency resulted in higher grain output than in the CT system (Biswakarma et al., 2021). Less machine trafficking and greater soil health may also contribute to higher grain output in ZT plots (Paul et al., 2014; Varatharajan et al., 2019a,b; Kumar et al., 2021, 2022), better root aeration (Choudhary et al., 2018; Singh et al., 2020a,b); and most notably, there will be less water stagnation after heavy rains and increased moisture conservation during dry periods (Choudhary et al., 2018, 2020; Kumar et al., 2022), a common feature during SW-monsoons in semi-arid IGPR.

P-fertilization had a considerable impact on maize and wheat grain and stover/straw yields, with P_50_+PSB+AMF+2FSP producing much greater grain and straw yields than the other treatments. P-fertilization has a direct effect on root growth and development, which increased vegetative and reproductive growth in relation to grain output (Rogério et al., 2013; Rana et al., 2018). As a result, yield in the P_50_+PSB+AMF+2FSP treatment was significantly higher than in the other treatments. In alkaline semi-arid IGPR soils, applied P combines with Ca and Mg ions to form Ca and Mg phosphates, rendering P inaccessible to plants (Kumar et al., 2015, 2016). As a result, 2 foliar sprays of P (2FSP) at the rate of 2% DAP in maize at KHS and PTS, as well as in wheat at TS and PFS, was found to be considerably beneficial for better N and P absorption through foliage, resulting in higher plant growth and photosynthetic activity, and increased grain yield (Rafiullah et al., 2017). Better P-solubilization and mobilization by the PSB and AMF inoculation along with P-fertilization led to higher P bio-availability and acquisition by the plants (Kumar et al., 2015, 2016). Thus, the combined application of P_50_+PSB+AMF+2FSP harnessed higher productivity which was followed by P_50_+PSB+AMF, P_50_+2FSP, and sole use of 100% P as soil applied-P, respectively (Suri and Choudhary, 2012; Rafiullah et al., 2017). The better maize yield under P_50_+PSB+AMF+2FSP over P_100_ alone might be attributed to the PSB+AMF’s synergistic effect on P-solubilization and mobilization (Suri et al., 2011, 2013), as well as increased P-absorption through foliar-P application (Rafiullah et al., 2017). As a result, foliar-P fertilization may have improved maize yield and PUE in this study (Rafiullah et al., 2017). Rafiullah et al. (2020) also found that grain yield is higher under combined application of P through soil and foliar means rather than soil-applied P alone.

Photosynthetic parameters were found to vary significantly in this study, with higher values in CA-based plots than in CT-based plots (Table 3). In CT-based FBCT–FBCT practice, the indicators of photosynthetic capacity (Pn, Gs, and Ci) were all significantly lower than in CA-based plots due to a higher stomatal limitation index. This phenomenon was also reported by Zeng et al. (2014). This ought to be due to the insufficiency of the leaf area. Because of the lower number of stomata compared to the higher photosynthetic demand, the reduced leaf area was unable to use the high accumulation of N and P in maize and wheat leaves. The primary cause of the decrease in Pn during this time was stomatal closure, which was associated with the senescence of older leaves, which further reduced the area and number of stomata. Furthermore, crop nutrient uptake and utilization is an important factor that affects physiological processes such as photosynthesis and yield generation (Choudhary et al., 2022). A significant link between yield and P content per unit leaf area has been established (Ciampitti and Vyn, 2012; Wang et al., 2020; Zhang et al., 2021, 2022). As a result, their photosynthetic capacity was temporarily reduced during the flowering stage. This was supported by the findings of Ma et al. (2021). The leaf transpiration rate and stomatal conductance were higher in the CA-based PRBZT–PRBZT system than in the CT-based FBCT–FBCT system. The lower stomatal limitation index in the CA-based system is due to the fact that residue-retained plots had a better microclimate due to better soil moisture in ZT plots compared to CT plots. Release of nutrients due to decomposition of residues under ZT acted as a substrate for greater microbial activity, resulting in better shoot and root growth (Choudhary and Behera, 2020). Better leaf area in CA-based CETMs during the flowering stage resulted in more efficient photosynthesis and dry matter accumulation, as well as more effective weed suppression. According to Yang et al. (2021), this was attributable to significant increases in root growth during the flowering stage of both maize and wheat, resulting in a greater foraging area for water and nutrient intake.

Photosynthetic indices (photosynthetic rate, stomatal conductance, transpiration rate, and efficiency) were significantly affected by P-fertilization techniques, with the greatest values under the P_50_+PSB+AMF+2FSP treatment. Since P is linked to improved root growth, it aids in the acquisition of all vital plant nutrients. As a result, P-applied plots grew faster than non-P plots (Rana et al., 2018). Improved root growth leads to increased food intake, photosynthates generation, metabolic activities, rapid cell division, and finally the formation of meristematic tissue (Kumar et al., 2015, 2016). Better meristematic tissues result in more active leaves per plant, which increases leaf area (Shevchuk et al., 2019). Higher leaf area and a lower stomatal limitation index suggest that stomata are not a limiting factor for photosynthesis when P_50_+PSB+AMF+2FSP is delivered by soil, foliar, and microbiological ways, resulting in a higher photosynthetic rate than when any one of the above techniques is used alone. The intercellular CO_2_ concentration was found to be higher in the control plot, showing that a non-stomatal factor, namely P mesophyll limitation, is the primary cause of the P_0_ treatment’s decreased photosynthetic rate. At flowering stage, phosphorus content per unit leaf area decreases under low P supply, resulting in lower photosynthetic activity. Additionally, this resulted in the partial closure of leaf stomata, which resulted in a considerable reduction in net photosynthetic rate. These findings are in conformity with Tao et al. (2022).

Higher uptake of grain P was observed in the PRBZT–PRBZT system in both maize and wheat crops during both study years. Higher grain yield due to enhanced soil physico-chemical and biological properties leading to enhanced P uptake in comparison to CT plots (Govaerts et al., 2007; Ram et al., 2011; Das et al., 2018; Ankit et al., 2022). Because of improved P-nutrition, root and shoot system, P solubilization and mobilization, and foliar-P supplementation, P-fertilization with PSB and AM-fungi versus foliar-P treatment had a significant impact on grain P uptake (Sobhana et al., 2012; Amanullah et al., 2016; Rafiullah et al., 2017; Varatharajan et al., 2019a). The P content (%) in maize and wheat grains and stover/straw at harvest was found to be higher in the PRBZT–PRBZT treatment and lowest in the FBCT–FBCT treatment. Similarly, the data from the current study revealed that varied CETM techniques had a considerable impact on P uptake, which followed the same pattern as that of nutritional content (Figure 7). Microorganisms degrading the crop residues result in mineralization of important plant nutrients for crop uptake and better SOC content in the soil, hence, leading to increased soil enzymatic activity, resulting in higher nutrient acquisition under the PRBZT–PRBZT treatment (Ram et al., 2011; Grzyb et al., 2020). In the PRBZT–PRBZT treatment, raised-beds (RB) coupled with ZT might have promoted the root development, resulting in a bigger foraging area for nutrient uptake, and as a result, with greater nutrient concentrations. Crop residues are nutrient storage areas; therefore their delayed and consistent release pattern boosted the nutrient availability for maize and the next wheat crop (Govaerts et al., 2007). Increased nutrient bio-availability again reduces the crop-weed competition for nutrient acquisition (Dass et al., 2016), resulting in enhanced dry matter accumulation and grain yield, and hence, nutrient uptake (Jinger et al., 2022). That’s why, ZT plots had higher nutrient uptake as a result of both higher nutrient concentration and higher yield as nutrient uptake is the function of both nutrient content (percent) and grain output (Zulfiqar et al., 2020; Dass et al., 2022).

The P-fertilization practices had a significant impact on P content (%) and uptake in maize and wheat grains and stover/straw at harvest during both years. Among the P-fertilization options, P_50_+PSB+AMF+2FSP resulted in the significantly largest nutritional content of P (%) and absorption. External application of P, combined with nutrient availability from the breakdown of agricultural waste, may have resulted in significant root proliferation, resulting in even better uptake of available nutrients from the soil (Kumar et al., 2016). Crop waste not only provided nutrients, but it also improved the soil’s physical, chemical, and biological properties (Choudhary et al., 2018, 2020). With enhanced crop growth the metabolic and photosynthetic activity of the plants will increase, resulting in improved nutrient absorption and assimilation by the crop (Qiao et al., 2019). Nutrient content tends to decrease as crop growth progresses, regardless of the fertilization method. It could be due to the nutrient concentrations being diluted as a result of increasing dry matter accumulation (Guo et al., 2020). A crop’s nutrient intake is determined by its age, crop type, soil type, soil nutrient status, variety, soil moisture, soil temperature, and other factors. According to Muneta et al. (2017) the nutrient uptake was shown to be higher under high fertility levels, which they ascribe to better nutrition and the buildup of nutrients in grains and stover/straw. Total nutrient uptake is a result of the product of yield and nutrient concentration (Rana et al., 2014).

The RUE and RWC were found to be significantly affected by both CETM and P-fertilization practices under CA-based MWCS. Compared to CT-based system, the CA-based ZT system showed improved RUE and RWC because of favorable planting geometry (raised-bed planting). This helped in capturing more solar energy due to better crop growth and canopy development with greater leaf area index, and hence, with more light interception and higher RUE (Varatharajan et al., 2019b). This geometry resulted in increased plant photosynthesis and competitive advantage over flat-bed sowing in terms of yield (Choudhary et al., 2018, 2020). The initial crop establishment and plant growth were better under the PRBZT–PRBZT system in MWCS due to the retention of crop residues at 6 t/ha per year on the soil surface, which reduced soil temperature while improving soil moisture content and resultant improved water uptake. In current study, all of these parameters may have significantly boosted the soil moisture retention with reduced evaporation, resulting in higher water productivity and RWC in the leaves (Paul et al., 2014). The P–fertilization practices also exerted a significant effect on RUE and RWC both for maize and wheat crop. Among P–fertilization practices, P_50_+PSB+AMF+2FSP resulted in significantly greater RUE and RWC than all other P-fertilization techniques. This may be accrued to the reason that the P is linked to greater root growth, which in turn improves the acquisition of all critical plant nutrients. The P-fertilization in combination with PSB and AMF, have a synergistic effect on P-availability owing to improved P-solubilization and mobilization, resulting in improved growth, light interception and RUE (Suri et al., 2011; Kumar et al., 2015, 2016). The P_50_+PSB+AMF+2FSP treatment imbedded with AM-fungi might have improved the plant’s water status by secreting growth-promoting hormones such as indole acetic acid, which promotes the expansion of the root system (Dilfuza, 2011; Kumar et al., 2016). Crop residue decomposition leads to improved soil building ability and its stability, which in turn boost the soil water retention (Leu et al., 2010; Humberto et al., 2015), This might have resulted in higher relative leaf water content and well developed root system in crop plants under double-ZT plots (Al-Khateb et al., 2018).

Pearson’s correlation, clustered heatmap, PCA, and SLMR analysis clearly revealed that the effective P-management under different CETMs led to improved photosynthetic characters, RWC, RUE and PAR interception. As a result, the grain yield increased both in maize and wheat under P_50_+PSB+AMF+2FSP coupled with double zero-tilled PRBZT–PRBZT system with crop residue retention at 6 t/ha per year. The association tests supported the idea that by integrating the soil applied-P, foliar-P nutrition, and microbial inoculants altogether resulted in positive P build-up and P bio-availability in the soil besides enhanced P acquisition by the maize and wheat plants (Rana et al., 2014; Choudhary et al., 2020). As a result, grain P uptake and yield had a strong correlation (Choudhary and Rahi, 2018; Kumar et al., 2021, 2022). The heatmap clustering proved the hypothesis that CETM and P–fertilization practices lead to improved photosynthetic indices, RUE, RWC, root dry weight, and P indices (Varatharajan et al., 2019a; Rajpoot et al., 2021). Heatmap clustering revealed that SLI, PHI, RDW, PCPULA, and TE were the primary contributors to grain yield, with SLI > PHI in maize and RDW > PCPULA > TE in wheat, respectively (Figure 9). This was reinforced by the PCA statistical technique, which demonstrated that photosynthetic and P indices had a significant impact on grain yield both in maize and wheat, whereas the SLI had a negative impact on grain productivity (Rana et al., 2014; Asmare and Markku, 2016). The SLMR analysis confirmed our prediction numerically, indicating that PUG, PAR @FL, and RDW all contributed to an increase in maize and wheat grain production to the tune of 73.26 and 56.55%; 9.73 and 10.83%; 4.80 and 25.03%, respectively; while PCPULA deterred the grain yield by 1.69 and 2.54% (Eqs. 9, 10 and Supplementary Table 4). As an outcome, our hypothesis was well proven that the integrated use of soil applied inorganic-P, foliar-P and microbial inoculants in conjunction with CA-based double zero-tilled PRBZT–PRBZT system, proved highly effective in enhancing the photosynthetic characteristics and crop productivity while ameliorating the nutrient and environmental stresses in maize and wheat in semi-arid Ustochrepts of the IGPR.

## Conclusion

In summary, the 2 years’ study indicated a clear positive impact of CA-based CETM and P-fertilization practices on photosynthetic indices with superior PAR interception, RUE, RWC, grain P uptake and grain yield of both maize and wheat crops under MWCS. The P-fertilization practice imbedded with soil applied-P, foliar-P and microbial inoculants (P_50_+PSB+AMF+2FSP) under CA-based double zero-tilled PRBZT–PRBZT system not only boosted the net photosynthetic rate but also had higher immediate water-use efficiency due to enhanced transpiration efficiency. The multivariate data analysis identified that the net photosynthetic rate was the vital factor in regulating the grain yield of both the test crops (maize and wheat). Likewise, double zero-tillage in MWCS also harnessed ∼13.2 and 14.9% higher grain yield in maize and wheat, respectively compared to CT-based FBCT-FBCT system. Overall, the CA-based double zero-tilled PRBZT–PRBZT system with crop residue retention at 6 t/ha per year, coupled with P_50_+PSB+AMF+2FSP may enhance the MWCS productivity along with resilience to environmental stresses in the south-Asian IGPR for ensuring sustainable food production in the region.

## Data availability statement

The original contributions presented in this study are included in the article/Supplementary material, further inquiries can be directed to the corresponding author/s.

## Author contributions

MH contributed to conceptualization, methodology formulation and implementation, resource, and review. AC contributed to conceptualization, project administration, methodology formulation and implementation, original draft preparation, reviewing, and Editing. IB contributed to data analysis and graphical works, editing and results validation. AD and SC contributed to editing of original and revised versions of the manuscript. GR, TV, and SD contributed to results compilation and draft preparation. VS contributed to graphs and maps preparation. RB, SG, and SK contributed to review of literature, basic analysis. PV, GK, and ED contributed to data collection and processing, and original draft preparation. MB and TB contributed to review of literature and results compilation. All authors contributed to the article and approved the submitted version.

## Abbreviations

AMF: arbuscular mycorrhizal fungi
CA: conservation agriculture
CETM: crop establishment and tillage management
CT: conventional tillage
DAP: Di-ammonium phosphate
DMRT: Duncan’s multiple range test
EC: electrical conductivity
FBCT: flat bed–conventional tillage
FBZT: flat bed–zero tillage
2FSP: two foliar sprays of phosphorus
ha: hectare
IGPR: Indo-Gangetic plains region
IRGA: Infrared Gas Analyzer
Ci: intercellular CO_2_ concentration
KHS: knee-high stage in maize
Tr: leaf transpiration rate
m ha: million hectares
mt: million tons
MT: maximum tillering stage
MWCS: maize–wheat cropping system
Pn: net photosynthetic rate
N: nitrogen
NDVI: normalized difference vegetation index
P: phosphorus
P_0_: no-phosphorus
P_50_: 50% recommended dose of P (as basal)
P_100_: 100% recommended dose of P (as basal)
PUE: phosphorus-use efficiency
PRBZT: permanent raised bed–zero tillage
PSB: phosphorus solubilizing bacteria
Parea: P content per unit leaf area
PAR: photosynthetically active radiation
PCA: principal component analysis
K: potassium
RBCT: raised bed–conventional tillage
RUE: radiation-use efficiency
RWC: relative water content
RWCS: rice–wheat cropping system
Sc: stomatal conductance
Ls: stomatal limitation index
t: tons
TS: tillering stage in wheat.
