# Supplementary material for: Double zero-tillage and foliar-P nutrition coupled with bio-inoculants enhance physiological photosynthetic characteristics and resilience to nutritional and environmental stresses in maize–wheat rotation
Source: Front Plant Sci. 2022 Sep 15;13:959541. doi: 10.3389/fpls.2022.959541 (PMC9520575; doi:10.3389/fpls.2022.959541)
Supplement: Supplementary file 1 [file Data_Sheet_1.PDF]

Supplementary Table 1

Influence of crop-establishment and tillage management (CETM) and P-fertilization practices on days taken to different phenological stages of maize and wheat under MWCS.

Treatments	Maize				Wheat	
	Days taken to 50% taselling		Days taken to 50% silking		Days taken to 50% flowering	
	2018-19	2019-20	2018-19	2019-20	2018-19	2019-20
**Crop-establishment and tillage management (CETM)**						
FBCT–FBCT	55.1a	54.6a	62.2a	60.4a	104.6a	104.1a
RBCT–RBZT	54.2a	53.7a	61.9a	59.7a	102.3ab	102.0ab
FBZT–FBZT	52.4a	51.3a	58.7a	57.9a	101.2ab	101.1ab
PRBZT–PRBZT	52.4a	51.2a	58.7a	56.4a	99.8b	99.9b
**P-fertilization practices**						
P_{100}	52.36cde	51.04bcd	58.41cde	57.53cde	104.8a	104.7a
P_{50}+2FSP	51.19de	50.89cd	57.15de	56.74de	103.3a	103.0a
P_{50}+PSB+AMF	54.07bc	53.26ab	60.64bc	59.18bcd	102.6a	102.4a
P_{50}+PSB+AMF+2FSP	50.48e	49.73d	56.47e	55.81e	98.2b	98.0b
P_0	57.22a	55.49a	63.51a	62.07a	105.8a	105.4a

[**Note:** M_1: Flat bed–conventional tillage (FBCT) both in maize & wheat; M_2: Raised bed–CT (RBCT) in maize & RB–zero tillage (RBZT) in wheat; M_3: FBZT both in maize & wheat; M_4: Permanent raised bed–ZT (PRBZT) both in maize & wheat. S_1- P_{100}:- 100% P as basal; S_2- P_{50} + 2FSP: 50% P as basal (P_{50}) + 2 foliar sprays of phosphorus (2FSP) as DAP (2%) at knee-high stage (KHS) and pre-tasseling stage (PTS) in maize and at tillering stage (TS) and pre-flowering stage (PFS) in wheat; S_3- P_{50} + PSB + AMF: P_{50} + PSB + AM-fungi (AMF); S_4- P_{50} + PSB + AMF + 2 FSP: P_{50} + PSB + AMF + 2FSP at KHS & PTS in maize and at TS & PFS in wheat; S_5- P_0: 100% N & K with no P (P_0) as control].

Supplementary Table 2

Results of cluster analysis of different photosynthetic indices, RUE, RWC, P-uptake in grains (PUG) under CETM and P-fertilization plots in maize and wheat.

**Vertical clusters**	**Photosynthetic indices, RUE, RWC, RDW, and P indices**	**Horizontal clusters**	**Treatments (CETM and P-fertilization plots).**
**In maize**			
• Outlier	ICO_2-C	• Outlier I	M2S1
• Cluster I	RUE, PR and TE	• Cluster I	MIS2,MIS3,M2S5,M4S5, M1S5, M3S5
• Cluster II	RDW, PAR@FL, RWC, TR and SC	• Cluster II	M1S4, M3S4, M4S2, M2S4 and M4S4
• Cluster III	PUG and PULA	• Outlier III	MIS1
• Cluster IV	P-HI and SLI	• Cluster IV	M4S3, M3S2,M3S3,M2S3, M4S1, M2S2 and M3S1
**In wheat**			
• Cluster I	P-HI and SLI	Cluster I	M4S5,M2S3,M2S5,M1S5 and M3S5
• Cluster II	ICO_2-C and RUE	• Cluster II	M1S3, M1S1 and M1S2
• Cluster III	PAR@FL, TR, RWC, SC, PUG, and PR	• Cluster III	M3S1, M2S1 and M2S4
• Cluster IV	RDW, TE and PULA	• Cluster IV	M3S2, M3S3,M3S4,M4S2, M4S1, M4S3,
-	-	• Cluster V	M4S4, M1S4 and M2S2

Supplementary Table 3

Results of factor loading PCA of photosynthetic indices, RUE, RWC, RDW and P-uptake in grains (PUG) of maize and wheat.

**Principal components**		**Maize**			**Wheat**		
		**PC 1**	**PC 2**	**PC 3**	**PC 1**	**PC 2**	**PC 3**
Initial Eigenvalues	Total	8.21	1.84	1.13	8.27	2.27	1.01
	% of Variance	63.13	14.14	8.73	63.62	17.50	7.78
	Cumulative %	63.13	77.28	86.00	63.62	81.11	88.89
Rotation Sums of Squared Loadings	Total	7.46	2.40	1.32	6.51	3.79	1.26
	% of Variance	57.37	18.49	10.14	50.05	29.16	9.69
	Cumulative %	57.37	75.86	86.00	50.05	79.21	88.89
				**Factor loadings[a]**			
**Eigen vectors[b]**				**Principal components**			
		**PC 1**	**PC 2**	**PC 3**	**PC 1**	**PC 2**	**PC 3**
P uptake by grain		**0.886**	0.113	0.379	0.871	0.251	0.319
PAR @flowering		0.807	0.233	-0.131	0.804	0.368	0.037
Photosynthetic rate		**0.955**	0.182	-0.103	**0.930**	0.293	0.133
Transpiration rate		0.871	0.166	-0.263	0.654	0.612	0.270
Transpiration efficiency		0.822	0.141	0.065	0.967	-0.023	0.000
Stomatal Conductance		0.844	0.348	-0.233	0.790	0.404	0.280
Root dry weight		0.839	0.125	-0.247	0.815	0.152	0.059
P-harvest index		-0.174	0.071	**0.962**	0.152	-0.036	**0.977**
Intercellular CO_2 concentration		0.187	**0.974**	0.026	0.137	**0.981**	-0.042
Stomatal limitation index		-0.201	-0.972	-0.059	-0.156	-0.974	0.054
P content per unit leaf area		0.862	-0.090	-0.159	**0.916**	0.071	0.007
Radiation use efficiency		0.790	0.358	-0.035	0.247	**0.869**	0.066
Relative water content		**0.887**	0.292	-0.044	0.788	0.522	0.143

Note: Extraction Method: Principal Component Analysis; Rotation Method: Varimax with Kaiser Normalization. [a]Boldfaced factor loadings are considered highly weighted; [b]Rotation converged in 5 iterations.

Supplementary Table 4

Stepwise multiple regression variance analysis of grain yield versus PAR interception, RDW and P indices of maize and wheat under MWCS.

	Source	DF	Seq SS	Contri-bution	Adj SS	Adj. MS	F-Value	P-Value	T-Value	Coef	SE Coef
**Maize grain yield**	**Regression**	4	4313586	89.48%	4313586	1078397	31.91	0.000	2.31	1253	542
	**PUG**	1	3531537	73.26%	434869	434869	12.87	0.003	3.59	78.5	21.9
	**PAR@FL**	1	468992	9.73%	219478	219478	6.49	0.022	2.55	1.193	0.468
	**RDW**	1	231508	4.80%	293843	293843	8.69	0.010	2.95	80.9	27.4
	**PULA**	1	81549	1.69%	81549	81549	2.41	0.141	-1.55	-12475	8031
	**Error**	15	506961	10.52%	506961	33797					
	**Total**	19	4820548	100.00%							
**Wheat grain yield**	**Regression**	4	5653959	94.95%	5653959	1413490	70.57	0.000	6.49	2393	369
	**PUG**	1	3367488	56.55%	961921	961921	48.02	0.000	6.93	102.9	14.8
	**PAR@FL**	1	644998	10.83%	113017	113017	5.64	0.031	2.38	0.766	0.323
	**RDW**	1	1490408	25.03%	1525802	1525802	76.17	0.000	8.73	42.26	4.84
	**PULA**	1	151066	2.54%	151066	151066	7.54	0.015	-2.75	-18433	6712
	**Error**	15	300463	5.05%	300463	20031					
	**Total**	19	5954422	100.00%							

Note: **S** = Standard error of the regression**, PRESS**= Predicted residual error sum of squares, **Seq SS** = Sequential sums of squares, **Adj SS** = Adjusted sum of squares, **Adj MS** = Adjusted mean squares.
